# Single-cell-derived ferroptosis signature predicts prognosis and therapy response in esophageal squamous cell carcinoma

**DOI:** 10.3389/fonc.2026.1873687

**Published:** 2026-07-17

**Authors:** Qin Xu, Yu Ma, Xuanqi Huang, Elyar abdukirim, Meng Yang, Chunli Song, Meng Liu

**Affiliations:** 1 School of Pharmacy, State Key Laboratory of Pathogenesis, Prevention and Treatment of High Incidence Diseases in Central Asia, Xinjiang Medical University, Xinjiang, Urumqi, China; 2 Affiliated Tumor Hospital of Xinjiang Medical University, State Key Laboratory of Pathogenesis, Prevention and Treatment of High Incidence Diseases in Central Asia, Xinjiang, Urumqi, China; 3 Department of Clinical Laboratory, The Fourth People’ Hospital of Urumqi, Xinjiang, Urumqi, China

**Keywords:** esophageal squamous cell carcinoma, ferroptosis, prognostic model, single-cell RNA sequencing, therapy

## Abstract

**Background:**

Esophageal squamous cell carcinoma (ESCC) is a highly aggressive malignancy with poor prognosis and limited therapeutic options. Ferroptosis, a regulated form of cell death characterized by iron-dependent lipid peroxidation, plays a crucial role in tumor progression and immune regulation.

**Methods:**

We integrated single-cell RNA sequencing (scRNA-seq) and bulk transcriptomic data to identify ferroptosis-active cellular subpopulations within the ESCC tumor microenvironment. A ferroptosis-related prognostic model was constructed using LASSO-Cox regression and validated across independent cohorts from TCGA and GEO. Associations with immune infiltration, tumor mutation burden, therapeutic response, and drug sensitivity were explored. Furthermore, functional experiments were conducted *in vitro* using the ESCC cell lines, and the four prognostic core genes were revalidated using an independent single-cell dataset, which was also fully confirmed in clinical ESCC tissue samples. In addition, Western blot analysis was performed to examine the expression levels of ferroptosis-related proteins following CDCA3 knockdown, and to further investigate the impact of CDCA3 depletion on the cellular response to the ferroptosis inducer RSL3.

**Results:**

Four ferroptosis-related genes (*CBS*, *CDCA3*, *GALNT14*, and *IDO1*) were identified used to construct a robust risk model, effectively stratifying patients into high- and low-risk groups with significant differences in survival, immune infiltration, and predicted treatment response. *In vitro* experiments confirmed that CDCA3 knockdown significantly inhibited the proliferation and migration of ESCC cells and induced ferroptosis. GSE188900 single-cell sequencing data further confirmed that the aforementioned genes were significantly upregulated at single-cell resolution in tumor cells, with consistent validation in clinical ESCC tissue samples, Moreover,experimental results showed that knockdown of CDCA3 lead to the downregulation of ferroptosis inhibitor-related genes and upregulation of ferroptosis-promoting genes, thereby enhancing the sensitivity to RSL3-induced ferroptosis.

**Conclusions:**

Our study presents a single-cell-resolved ferroptosis gene signature with strong prognostic and therapeutic implications for ESCC. The signature was validated in clinical tissue samples, and this model lays the foundation for ferroptosis-targeted therapeutic strategies.

## Introduction

Esophageal squamous cell carcinoma (ESCC) is among the most aggressive and prevalent malignancies of the upper gastrointestinal tract, with a particularly high incidence and mortality rate in East Asia (1–3). Despite advances in early detection techniques such as high-definition endoscopy and improvements in multimodal therapies—including surgery, radiotherapy, chemotherapy, and immune checkpoint inhibitors—more than 60% of patients are diagnosed at an advanced stage, and relapse or therapeutic resistance often occurs rapidly (4–6). Traditional TNM staging and histopathological classifications are insufficient for guiding personalized treatment decisions, underscoring the urgent need for robust molecular prognostic models tailored to individual patient biology (7).

Ferroptosis is a distinct form of regulated cell death characterized by iron accumulation and lipid peroxidation (8). Emerging evidence suggests that ferroptosis plays a critical role in tumor suppression, drug sensitivity, and immune activation (9, 10). In ESCC, ferroptosis has been shown to influence both tumor progression and the immune landscape by reshaping the tumor microenvironment. The dynamic regulation of ferroptosis markers—such as *GPX4* and *SLC7A11*—has been associated with immune resistance and poor prognosis (11, 12).

To address the current gap in individualized prognostication and therapy guidance, we applied single-cell transcriptomics and bulk sequencing data to develop a novel ferroptosis-based prognostic model. By leveraging advanced computational methods, including pseudotime trajectory analysis, immune profiling, and machine learning, we identified a collection of single-cell ferroptosis-related gene signatures that show significant associations with survival outcomes and therapeutic responses. The proposed model was validated across multiple cohorts and supported by *in vitro* experimental evidence, demonstrating both scientific robustness and clinical translational value.

## Materials and methods

### Single-cell RNA sequencing data organization

Single-cell datasets retrieved from the GEO database (https://www.ncbi.nlm.nih.gov/geo/) were directly imported into Seurat V4 for downstream analysis. Only cells with gene expression counts between 200–6000 and mitochondrial gene percentages below 10% were analyzed. Single-cell gene expression profiles were preprocessed, downscaled by principal component analysis (PCA), and the batch effect of different samples was mitigated using the Harmony method. A total of 22 cell clusters were generated in Seurat using t-SNE at a resolution of 0.8, and cell type-specific transcriptional profiles were characterized through cluster-based differential expression analysis (Find All Markers function). Subsequent annotation using established lineage markers enabled systematic classification of cellular subpopulations, followed by quantitative assessment of their relative abundance across samples.

### Ferroptosis-related gene scoring based on single-cell clusters

The enrichment scores of 484 ferroptosis-related genes were calculated using the AUCell package. Using the “AUCell explore Thresholds” algorithm, we established optimal cutoff values for detecting cells with significant pathway activity. Subsequent visualization of single-cell AUC scores through t-SNE dimensional reduction enabled clear identification of transcriptionally active subpopulations within the dataset.

### Pseudotemporal trajectory analysis

Developmental trajectories were reconstructed using Monocle2, selecting highly dispersed genes for branch-dependent expression modeling.

### Cell communication analysis

Cell-cell communication analysis was performed using CellChat. CellChat objects from each group were merged with the “mergeCellChat” function, the total number of interactions and their intensity were compared, and the results were visualized using the “netVisual” family of functions.

### Pathway enrichment analysis

GO enrichment analysis was performed using the “clusterProfiler” R package (version 4.2.2) to explore the biological functions and pathways of differential gene sets in subpopulations of ESCC cells with high ferroptosis activity. Gene set enrichment analysis (GSEA) was conducted using the MSigDB c2 molecular signatures to explore the biological functions of correlated DEGs. For each comparison, 1000 permutations were per-formed on log2FC-ranked gene lists, with the significance threshold set at p<0.05. Pathway activity was further evaluated through gene set variation analysis (GSVA), with results visualized via the pheatmap package in R.

### Construction and evaluation of a prognostic model for ferroptosis genes

The correlation between differentially expressed genes associated with ferroptosis at the single-cell level and overall survival (OS) was evaluated by combining transcriptomic data from 125 cases as a training set, TCGA data as a validation set and GSE53624 as an independent validation cohort. First, univariate Cox regression (*p* < 0.05) screened ferroptosis-related genes for overall survival (OS). A LASSO Cox model was then built with the glmnet package (R v4.2.2). Optimal tuning was achieved by ten-fold cross-validation with λ = 0.05; prior to cv.glmnet the data were split 7:3 into training and internal-test sets. Genes retaining non-zero coefficients were retained as predictors. Patient-specific risk scores were computed with RiskScore = 0.1825 * *CBS* + 0.1609 * *CDCA3* + 0.1284 * *GALNT14* + 0.1465 * *IDO1* and used to stratify patients into low- and high-risk groups. We evaluated risk models and clinicopathologic characteristics by using univariate and multivariate Cox regression analyses. We employed the R “RMS” package to develop a validated nomogram for multi-temporal overall survival prediction.

### Immune infiltration and TMB in a genetic model of ferroptosis

Immune cell expression profiles were obtained from TISIDB and quantified for 22 cell types across 125 ESCC samples using ssGSEA (GSVA package). We download mutation data for 95 ESCC patients from the TCGA database and processed them with the maftools package to depict variant spectra (SNVs and indels), tumour-mutational burden and variant-allele frequencies.

### Drug sensitivity analysis

Drug sensitivity profiles (IC50 values) and matched transcriptomic data were obtained from GDSC (Genomics of Drug Sensitivity in Cancer). OncoPredict (v0.2) was used to estimate the median inhibitory concentration (IC50) of chemotherapeutic agents via ridge regression, enabling prediction of drug response in the 125 ESCC patients stratified by risk status.

### Esophageal squamous cell carcinoma cell culture

All ESCC cell lines used in this study were derived from the Shanghai Institute of Cell Biology, Chinese Academy of Sciences.These cell lines were authenticated by short tandem repeat(STR) profiling to confirm their identity and ensure no cross-contamination. Additionally,routine testing confirmed that all cell lines were free of mycoplasma contamination.These cells were cultured in RPMI 1640 medium supplemented with 10% fetal bovine serum (FBS) and 1% penicillin/streptomycin (double antibiotics). The cells were maintained in a cell incubator set at 37°C, with 95% humidity and 5% CO_2_ concentration, to ensure optimal growth and proliferation conditions.

### *CDCA3* transfection

The cells were evenly seeded into a 6-well plate. When the cells reached 50% confluence, three siRNA sequences purchased from Beijing Heyuan Biotechnology Co. Ltd. were formulated into complexes with Lip2000 and added to cells. After 6 hours, the medium was replaced, and the cells were allowed to continue growing. The siRNA sequences are detailed in the **Supplementary Materials**.

### Western blot

The cells were collected and lysed with lysis buffer on ice. The lysates were then centrifuged at 12000g for 15 minutes at 4 °C to obtain the protein supernatant. After quantifying the protein concentration using the BCA method, the samples were prepared and subjected to electrophoresis. Following electrophoresis, the proteins were transferred to a membrane, which was then blocked. The membrane was incubated with the primary antibody overnight. On the second day, the secondary antibody was added and incubated at room temperature for 2 hours, followed by color development.

### CCK8 assay

The cells were seeded evenly into a 96-well plate at a density of 3×10^4^ cells per well. After the cells had adhered to the plate, 10 µL of CCK-8 solution was added to each well, and the plate was incubated in the cell culture incubator for 2 hours. Subsequently, the cell viability was measured at 0 hours, 24 hours, 48 hours, and 72 hours using a microplate reader at a wavelength of 450 nm.

### EDU assay

Cells were seeded evenly into a 24-well plate at a density of 5×10^4^ cells per well. After edu labeling, the cells were fixed with a fixative solution. Following fixation, the cells were treated with a permeabilization solution for 15 minutes. Subsequently, the cells were stained for proliferation and incubated in the dark for 30 minutes. After staining, the cells were washed with a washing solution and then stained with hoechst for nuclear staining for 10 minutes. Finally, the cells were imaged using a fluorescence microscope.

### Transwell assay

The matrix gel was diluted at a ratio of 1:8 and added to the culture insert, which was then incubated in the cell culture incubator for 2 hours. Subsequently, 5×10^4^ cells were seeded into the upper chamber of the insert, while the lower chamber was filled with 20% complete culture medium. After 24 hours, the cells were fixed, stained, and photographed. The migration assay was performed in the same manner as the invasion assay, but without the addition of matrix gel.

### Quantitative real-time polymerase chain reaction (qRT-PCR)

Total cellular RNA was extracted using an RNA extraction kit, and reverse transcription was performed to obtain cDNA using Wuhan Sevier Reverse Transcription Reagent (GM2005). reverse transcription reaction conditions were 25 °C for 5 min, 42 °C for 30 min, and denaturation at 85 °C for 5 s, followed by cooling to 4 °C. Quantitative polymerase chain reaction (RT-qPCR) was performed using a real-time fluorescence quantitative PCR instrument. Thermal cycling conditions consisted of: 95 °C for 30 sec (initial denaturation), followed by 40 cycles of 95 °C for 15 sec and 60 °C for 30 sec. β-actin (F:5’-CTCCATCCTGGCCTCGCTGT-3’; R:5’-GCTGTCACCTTCACCGTTCC-3’) served as the endogenous control, and the target gene primers can be found in the **Supplementary Materials**. quantitative analysis was performed by calculating the 2 -ΔΔCt value.

### Fe^2+^ assay

The cells were adjusted to a concentration of 1×10^7^/mL, and then 1 mL of extraction buffer was added. The mixture was subjected to ultrasonic disruption. Subsequently, the samples were centrifuged at 10000g for 10 minutes at 4 °C, and the supernatant was collected. Meanwhile, the standard was diluted in a gradient of concentrations to plot a calibration curve. Finally, the absorbance of the supernatant was measured at 593 nm, and the concentration was calculated using the calibration curve formula.

### Malondialdehyde assay

The cells were adjusted to a concentration of 5×10^6^ cells/mL, and then 1 mL of extraction buffer was added. The mixture was subjected to ultrasonic disruption. Subsequently, the samples were centrifuged at 8000g for 10 minutes at 4 °C, and the supernatant was collected. The supernatant was then mixed with the MDA assay working solution and incubated in a water bath at 100 °C for 60 minutes. After incubation, the samples were centrifuged again at 10000g for 10 minutes at room temperature. Finally, the absorbance was measured at 532 nm and 600 nm, and the results were calculated using the appropriate formula.

### Glutathione assay

The cells were adjusted to a concentration of 5×10^6^ cells/mL, and then 1 mL of Reagent 1 was added. The mixture was subjected to repeated freeze-thaw cycles (2–3 times) in liquid nitrogen. Subsequently, the samples were centrifuged at 12000g for 10 minutes at 4 °C, and the supernatant was collected. Meanwhile, the standard was diluted in a gradient of concentrations. The samples were mixed with Reagents 2 and 3, then allowed to stand for 2 minutes. The absorbance was measured at 412 nm, and the results were calculated using the appropriate formula.

### Lipid ROS assay

First, remove the culture medium from the cells and add an appropriate amount of diluted DCFH-DA solution. Then, incubate the cells in a cell culture incubator at 37 °C for 1 hour. After incubation, wash the cells three times with serum-free culture medium to remove unbound DCFH-DA. Finally, observe the cells using a fluorescence microscope or detect them using a flow cytometer with an excitation wavelength of 488 nm and an emission wavelength of 525 nm.

### Statistical analysis

Continuous variables were analyzed with the Wilcoxon rank-sum test, while categorical variables were evaluated using χ² or Fisher’s exact tests. Survival outcomes were visualized via Kaplan-Meier curves (generated with the survminer R package) and compared using log-rank testing. Feature selection and prognostic modeling employed LASSO-Cox regression, with predictive accuracy quantified by area-under-curve (AUC) metrics from ROC and time-dependent ROC analyses. All tests were two-tailed (significance threshold: *P* < 0.05), implemented in R v4.2.2.

## Results

### Identification of ferroptosis-related subpopulations

**Figure 1:**
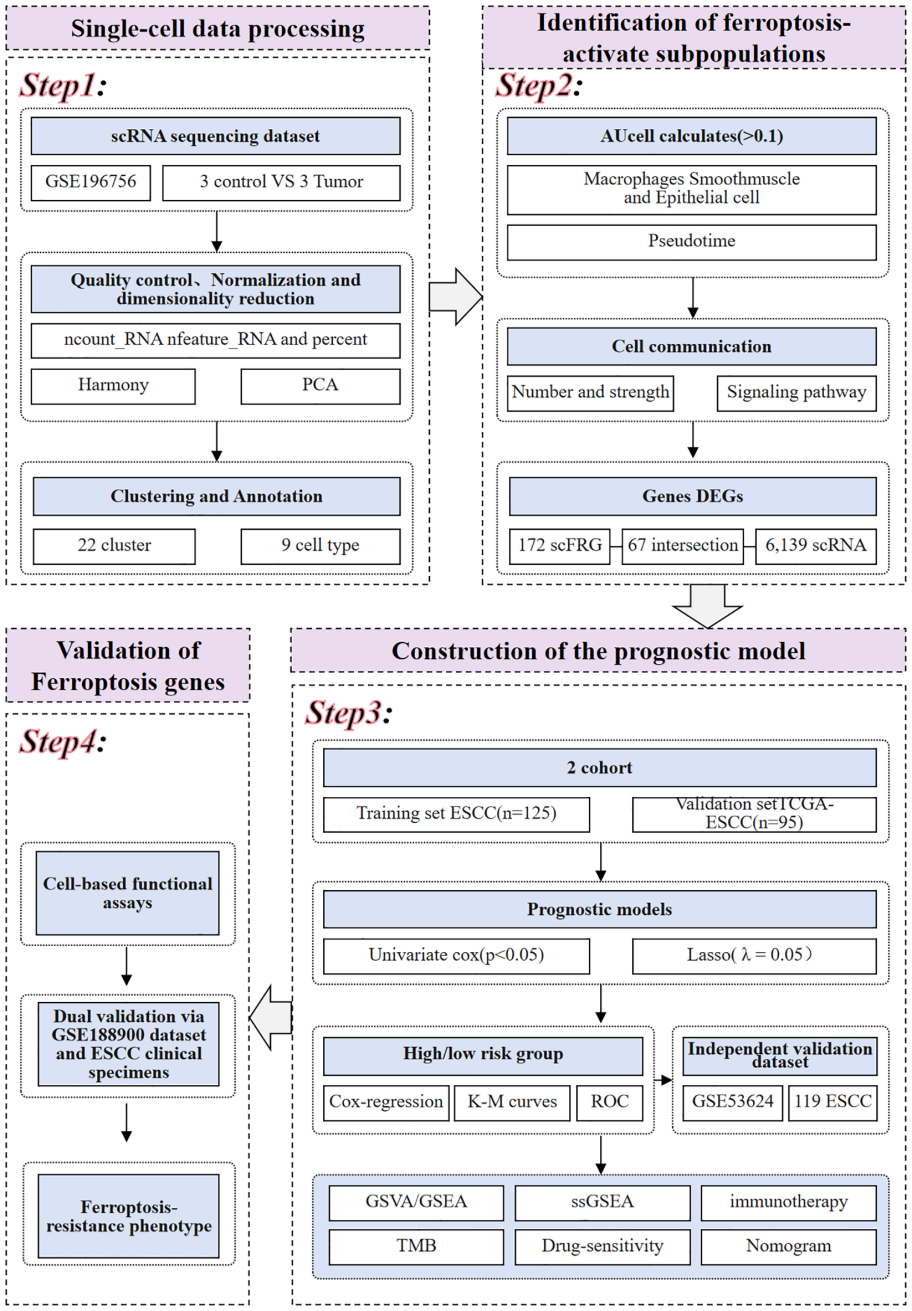
The flowchart of this study.

**Figure 2:**
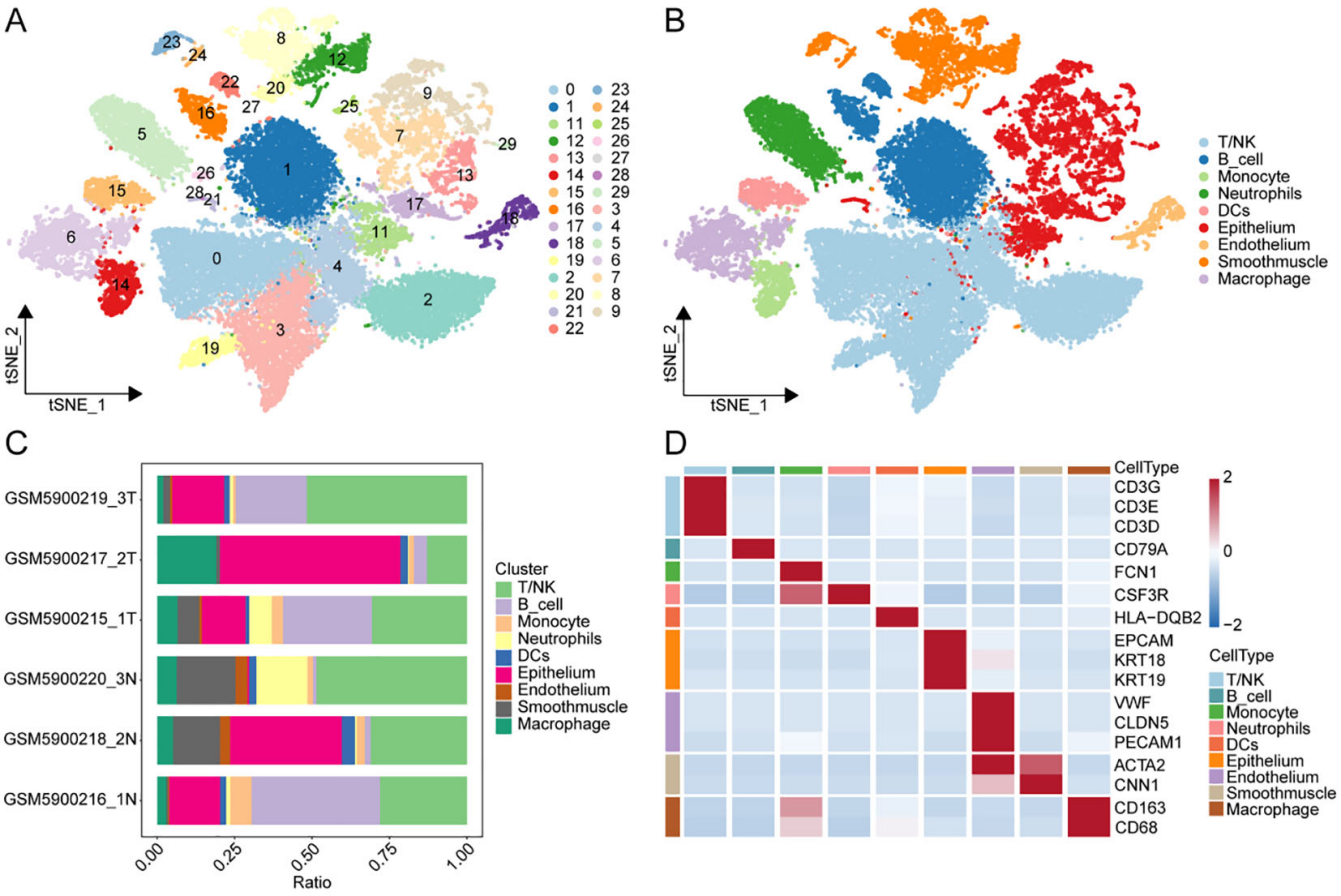
Single-cell data processing. **(A)** The t-SNE plot reveals the distribution of ESCC cell clusters. **(B)** The t-SNE plot shows the annotation results of ESCC cell subclusters. **(C)** The histogram presents the differences in cell type distribution between ESCC and normal control groups. **(D)** The expression of marker genes in different cell types is shown.

**Figure 3:**
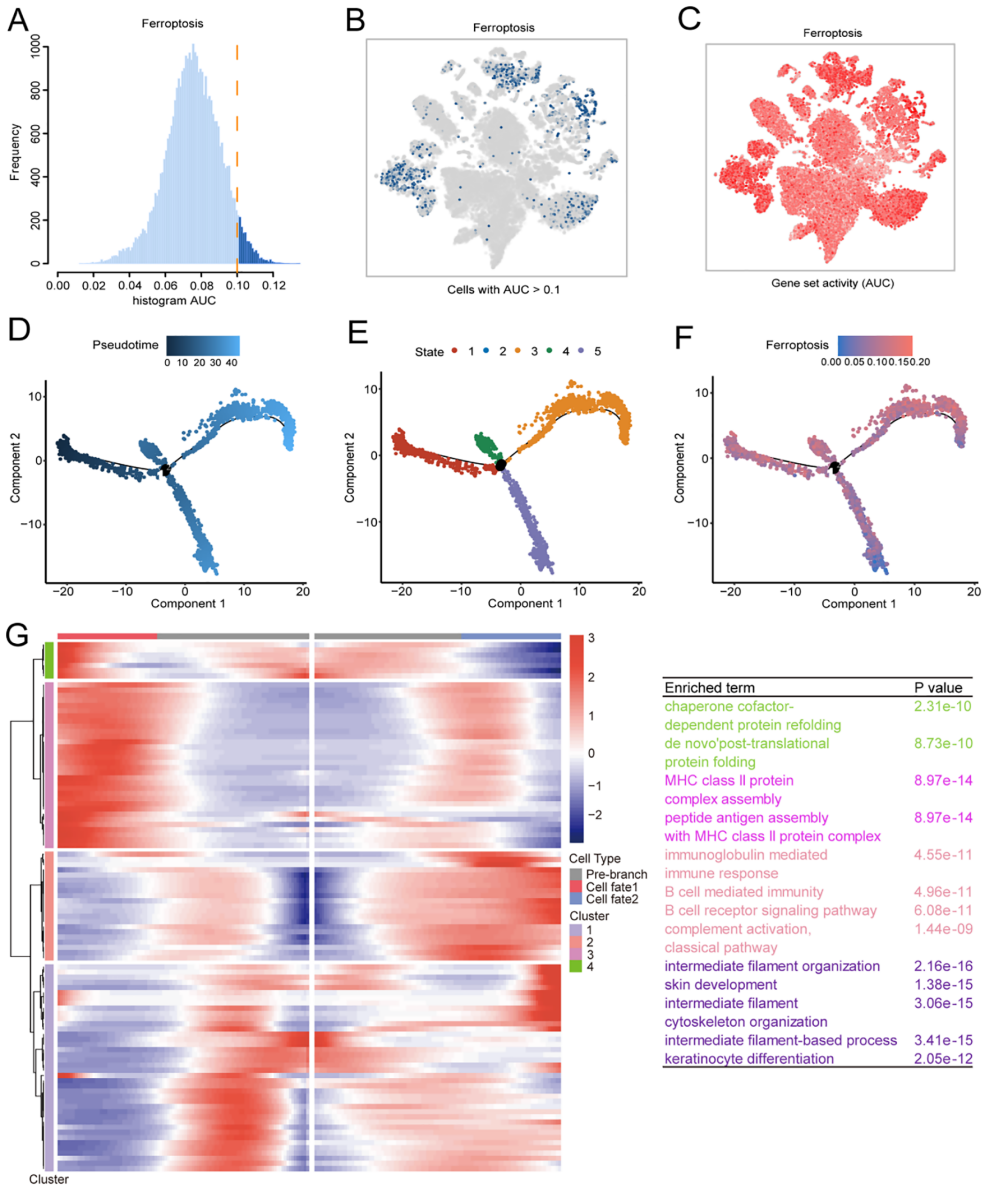
Cell annotation and identification and developmental trajectory analysis of ferroptosis-activated epithelial cells, macrophages, and smooth muscle cells. **(A)** The AUC score threshold for ferroptosis-related genes is set at 0.1. **(B)** The t-SNE plot displays the distribution of cells with AUC values exceeding the threshold. **(C)** The t-SNE color plot reflects cell activity scores, with brighter colors indicating higher cell activity. **(D)** The pseudotime trajectory color gradient transitions from dark blue to light blue. **(E)** Through Monocle2 analysis, the pseudotime trajectory is divided into five different states. **(F)** In the pseudotime trajectory plot based on AUCell scores, darker colors indicate higher ferroptosis activity. **(G)** The heatmap reveals differentially expressed genes in different cell fate branches and the enriched GO pathways of gene clusters.

**Figure 1** illustrates the overall workflow of the research presented in this paper. Following quality control and doublet removal, 34,724 cells were retained from the GSE196756 ESCC single-cell dataset. A total of six samples were included in this study, comprising three cancer and three control samples, with relatively uniform cell distribution between groups and no obvious batch effect. Based on gene expression characteristics, cells were clustered into 22 subpopulations and annotated by cell-specific biomarkers into 9 cell types: T/NK cells, B cells, monocytes, neutrophils, dendritic cells, epithelial cells, endothelial cells, smooth muscle cells and macrophages (**Figures 2A, B**). A heatmap displays the cellular composition across samples, and the specific genes associated with each cell type are visualized (**Figures 2C, D**). These findings established a cellular atlas of ESCC and identified key subpopulations for further analysis. To screen for subpopulations of cells with ferroptosis activity at the single-cell level, the study stratified cells based on an optimized threshold of ferroptosis-related gene expression (AUC > 0.1), and cells exceeding this threshold were defined as ferroptosis active cells (**Figure 3A**). The results showed that a total of 416 cells possessed significant ferroptosis activity. Further analysis showed that macrophages, epithelial cells, and smooth muscle cells displayed the highest ferroptosis activity (**Figures 3B, C**). Based on pseudotime trajectory analysis, the dynamic transformation of these three types of cells during ferroptosis was revealed, in which ferroptosis activity was markedly elevated in state 3 and showed a declining trend in state 5 (**Figures 3D–F**). Differential expression analysis performed before and after branching points further revealed the gene regulatory network in the critical process of ferroptosis. In the track branches, different pathways showed regional expression patterns: protein folding and assembly, cytoskeleton and organization pathways were highly enriched in the anterior branch, and immune response and complement activation pathways were predominantly expressed in branches Cell fate2 and Cell fate1, respectively, revealing their potential roles in cell fate differentiation (**Figure 3G**). This indicated dynamic ferroptosis regulation among different ESCC cell types, revealing critical differentiation trajectories.

### Ferroptosis activates intercellular communication between specific immune subpopulations of cells

**Figure 4:**
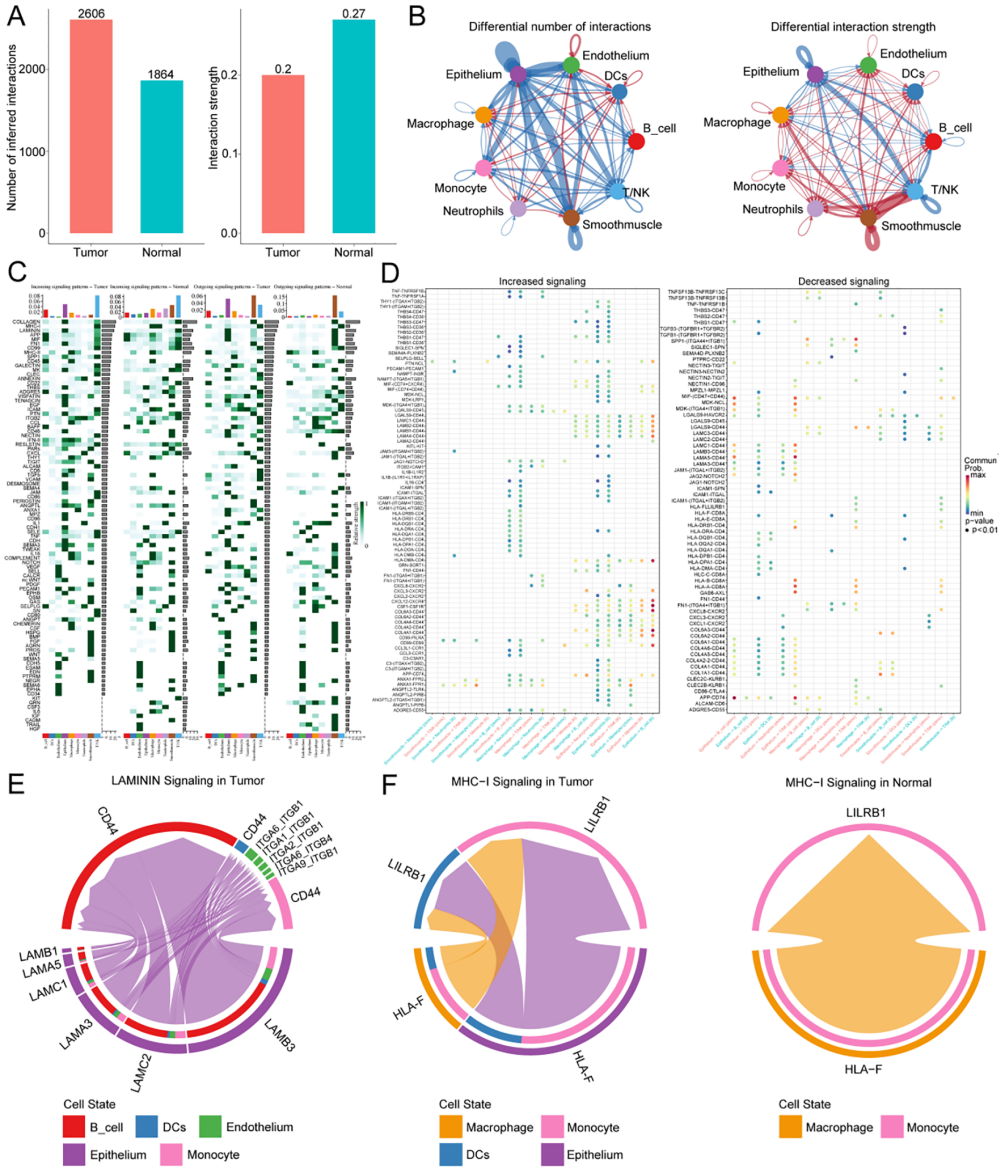
Analysis of inter-cellular communication among epithelial cells, macrophages, and smooth muscle cells in ESCC. **(A)** Analysis of the number and strength of interactions between normal cell types and ESCC cell types. **(B)** A network diagram illustrates the changes in the number and strength of interactions between normal cell types and ESCC cell types. **(C)** Signal pathways involved in the reception and transmission of signals between normal cells and ESCC cells. **(D)** Strength of communication between epithelial cells, macrophages and smooth muscle cells and other cell subpopulations characterised by an increase or decrease in receptors. **(E)** Receptor pairs related to the LAMININ signaling pathway’s incoming and outgoing signals with ESCC cells. **(F)** Receptor pairs related to the MHC-I signaling pathway’s incoming and outgoing signals with ESCC cells.

In this study, the CellChat algorithm was used to systematically analyze the cellular interactions in the ESCC tumor microenvironment, and it was found that com-pared with normal tissues, the intensity of cellular communication in tumor samples was significantly elevated, and a unique bidirectional regulatory network was formed (**Figures 4A, B**). At the signaling pathway level, LAMININ and MHC-I signaling exhibited more complex and active intercellular interactions in tumor samples; LAMININ signaling pathway mainly involved bidirectional interactions among epithelial cells, monocytes, and endothelial cells, whereas MHC-I signals were dominated by epithelial cells in tumor tissues and transmitted signals to immune cells (e.g. macrophages and dendritic cells) to form a unique immunoregulatory network (**Figures 4C, D**). In contrast, MHC-I signaling appeared comparatively simple and attenuated in normal tissues. These results suggest that LAMININ and MHC-I signaling pathways are abnormally activated in the ESCC tumor environment, which could critically influence tumor-immune cell crosstalk (**Figures 4E, F**). These results highlighted ferroptosis-induced alterations in intercellular signaling, particularly involving immune subpopulations.

### Functional enrichment analysis of DEGs associated with ferroptosis-activated cells

**Figure 5:**
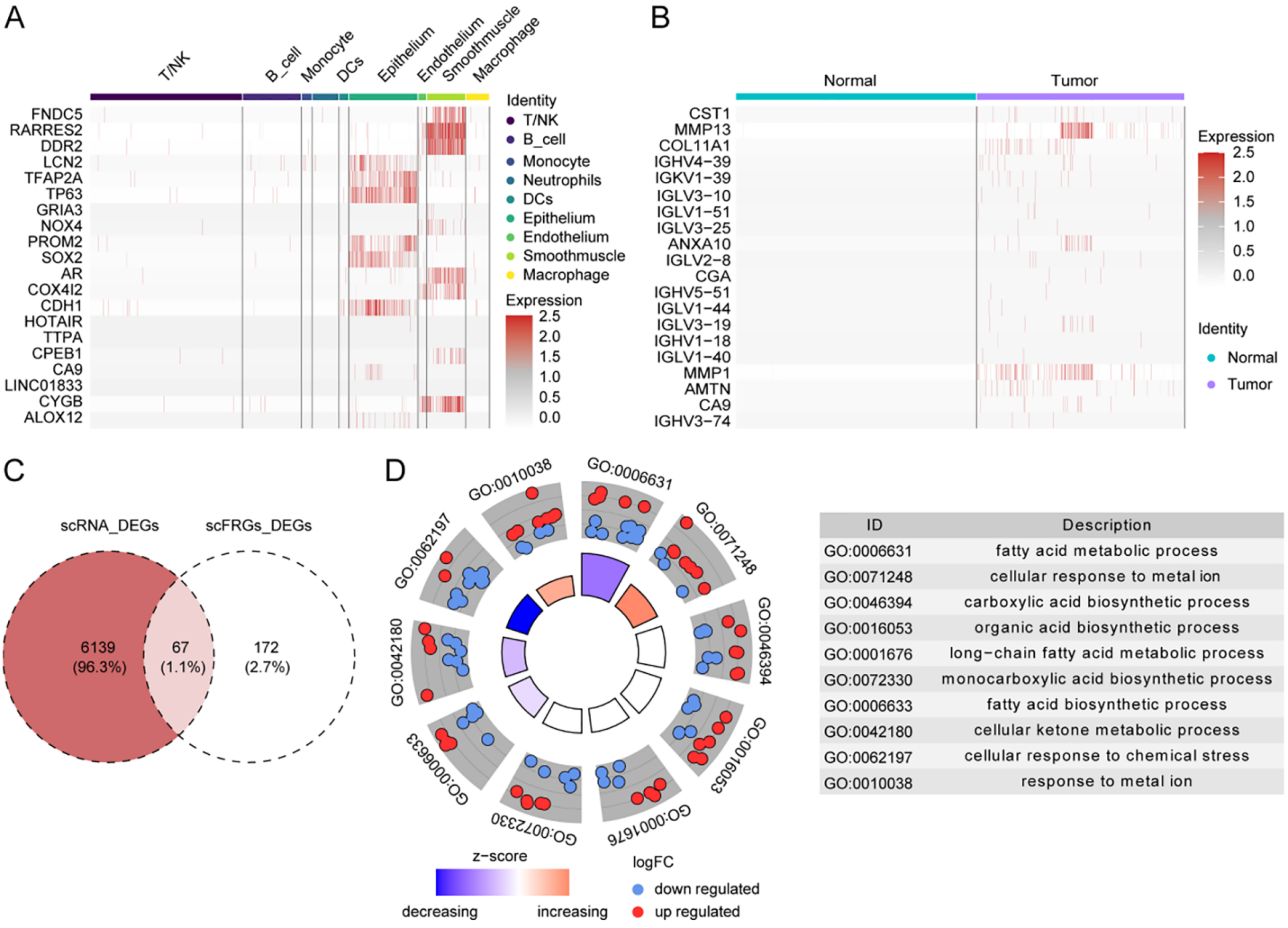
Visualization of functional enrichment analysis results. **(A)** Significance analysis of the top 20 differentially expressed genes (DEGs) between ESCC and control samples. **(B)** Differential gene expression in ferroptosis-activated epithelial cells, macrophages, and smooth muscle cells in ESCC. **(C)** Venn plots demonstrating the intersection of the key genes. **(D)** Gene Ontology enrichment analysis of key genes.

To explore differentially expressed genes (DEGs) and their functions in ferroptosis-active cells (macrophage, epithelial and smooth muscle), 172 DEGs were screened, and heatmaps were generated based on significance thresholds (adjusted P < 0.05, log2FC > 0.25 or < –0.25) to illustrate the expression patterns of the top 20 genes. These genes included *FNDC5*, *RARRES2*, *DDR2*, *LCN2*, *TP63*, *GRIA3*, *NOX4*, *SOX2*, and *CA9* (**Figure 5A**). By comparison with normal control samples, 6,139 DEGs were identified, of which the top 10 up-regulated genes (e.g. *MMP3*, *COL11A1*, *CA9*, etc.) and down-regulated genes (e.g. *CST1*, *ANXA10*, etc.) were visualized and presented (**Figure 5B**). Venn diagram-based intersection analysis identified 67 core hub genes that were consistently co-expressed across three ferroptosis-active cell types and ESCC tissues, and GO analysis showed their involvement in fatty acid metabolism, metal ion response, carboxylic acid biosynthesis and other biological processes (**Figures 5C, D**). These enriched biological processed underscore ferroptosis’s role in metabolism, immune modulation, and tumor progression.

### Construction and validation of a genetic scoring model for ferroptosis risk at the single-cell level

**Figure 6:**
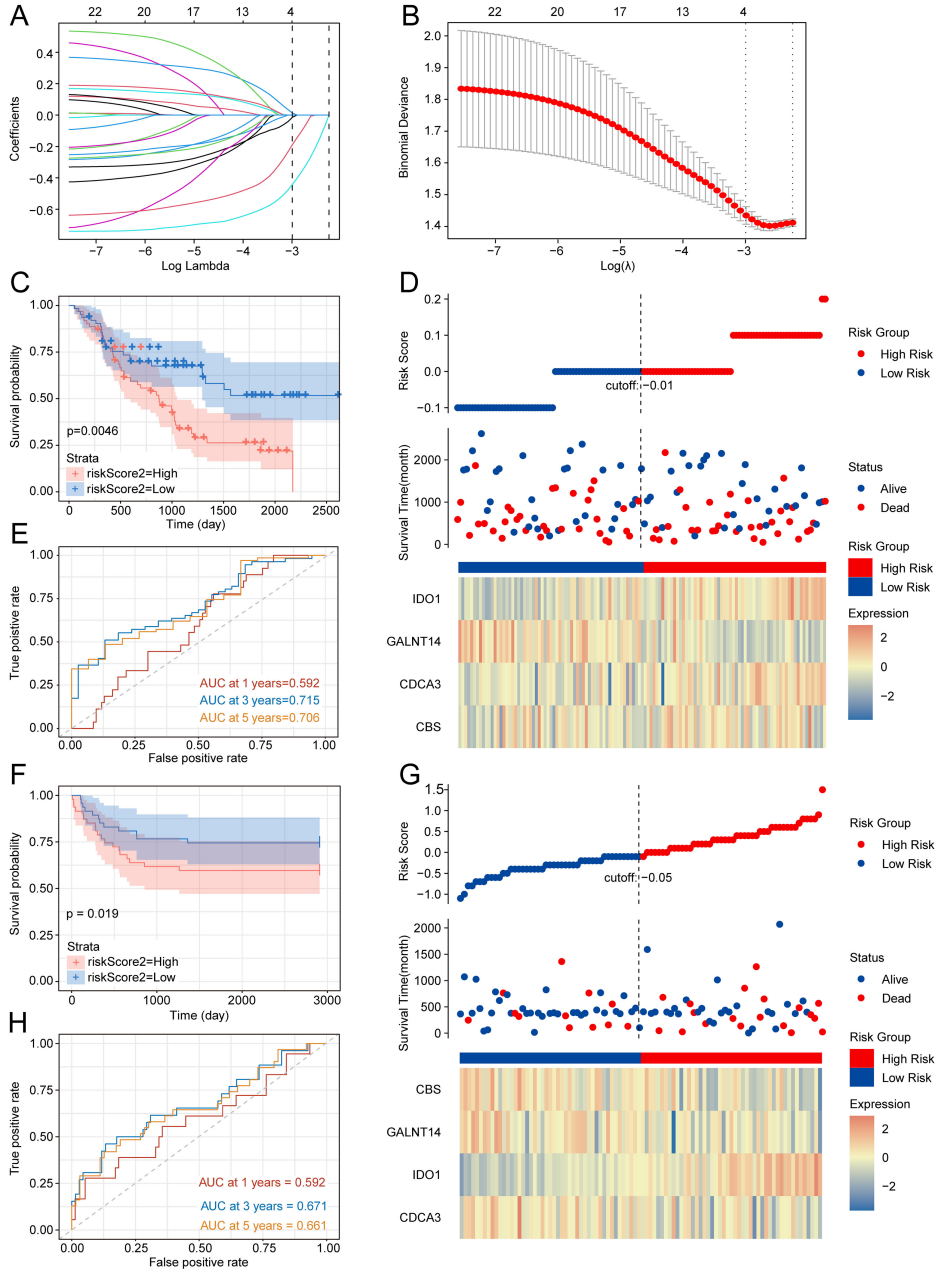
Identification of core genes involved in ferroptosis. **(A)** The trajectory plot of variable changes in Lasso regression **(B)** The confidence intervals corresponding to each lambda value in Lasso regression. **(C)** The survival curves of high-risk and low-risk patients in the training cohort. **(D)** The risk ternary plot of the training set, showing the distribution characteristics of risk. **(E)** The dynamic ROC curves of the model for 1-year, 3-year, and 5-year survival in the training cohort. **(F)** The survival curves of high-risk and low-risk patients in the validation cohort. **(G)** The risk ternary plot of the validation set, showing the distribution characteristics of risk. **(H)** The dynamic ROC curves of the model for 1-year, 3-year, and 5-year survival evaluated in the validation cohort.

In order to identify genes associated with prognosis-related traits in a central set of 67 genes specific to the transcriptome data of 125 ESCC patients, four prognosis-associated genes significantly associated with prognosis were identified by univariate Cox analysis. Subsequently, LASSO regression was applied in the training set (ESCC, n=125), the validation set (TCGA, n=95) and the independent sample set GSE53624 (ESCC, n=119) to construct a risk prediction model containing the 4 genes (**Figures 6A, B**). Using risk score stratification, patients were divided into high- and low-risk cohorts with significantly distinct overall survival outcomes (log-rank P < 0.05). The model demonstrated robust prognostic performance, with time-dependent AUCs of 0.592 (1-year), 0.715 (3-year), and 0.706 (5-year) in the training cohort, and 0.592, 0.671, and 0.661 (**Figures 6C–H**), respectively, in the validation cohort (**Supplementary Figures 1A–C**). In addition, to explore the mechanistic basis of FRG-mediated fractional-risk modulation among DEGs, we conducted gene set enrichment analysis (GSEA) leveraging pathway annotations from MsigDB. GSEA enrichment analysis revealed that the high-risk group was significantly enriched for pathways such as human cytomegalovirus infection, ribosomes, PI3K-AKT signaling, adhesion patches, and ECM-receptor interaction (**Supplementary Figures 2A–F**). GSVA analysis further revealed the functional significance of these pathways, suggesting that they may drive ESCC progression and provide a basis for targeted therapy (**Supplementary Figure 2G**). These pathway alterations might contribute to disease progression and represent potential therapeutic targets.

### Comparison immune infiltration between risk subgroups associated with ferroptosis genes

**Figure 7:**
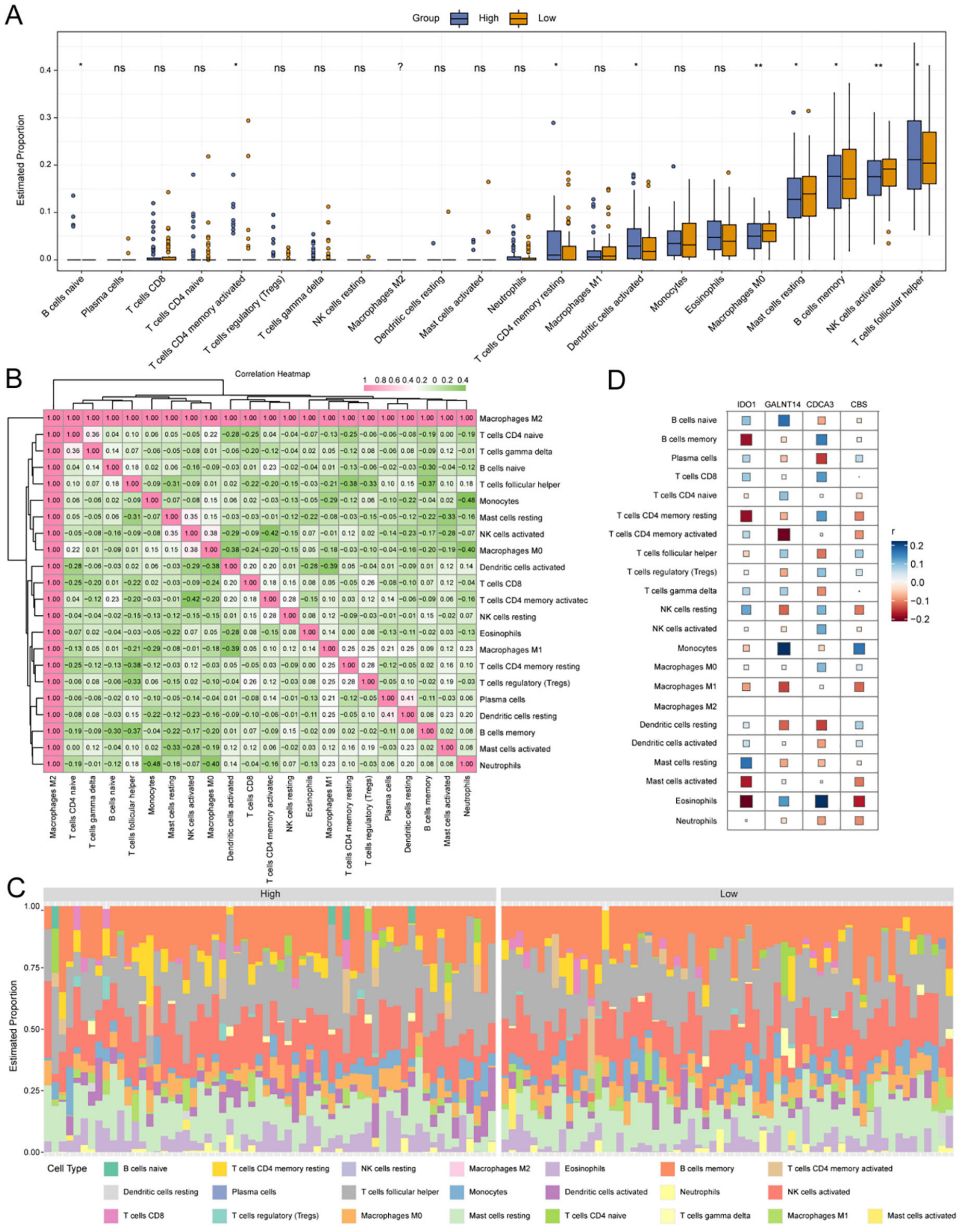
Characteristics of immune infiltration and tumor mutation burden analysis in high and low risk groups. **(A)** Expression patterns of 22 immune cell types in high-risk and low-risk groups. **(B)** Heat map of correlation between immune cells. **(C)** Proportion of immune cell types in high-risk and low-risk groups. **(D)** Heat map of correlation between ferroptosis markers and immune cells. *: P<0.05; **: P<0.01; ns: not significant (no statistically significant difference, P≥0.05).

To further assess the immune cell infiltration in ESCC tumor tissues, the relative abundance of immune cell subpopulations was quantified using the CIBERSORT algorithm. The results showed that most of the immune cell subtypes exhibited significant differences between the high and low expression groups. Specifically, CD4 memory activated T cell, CD4 memory resting T cell, activated dendritic cells, macrophages M0, resting mast cells, memory B-cell, activated NK cells, and follicular helper T-cell were significantly enriched in the high-risk group. (**Figures 7A, C**). Correlation heatmaps further revealed an intricate network of correlations between different immune cell subpopulations, especially between T cells and macrophages, which showed a strong positive correlation, suggesting their possible synergistic role in the tumor immune microenvironment (**Figure 7B**). In addition, heatmaps showed that *CBS* was negatively correlated with macrophages M0 and mast cells activated, *IDO1* was negatively correlated with a variety of immune cell subpopulations, and *CDCA3* showed an overall trend of positive correlation (**Figure 7D**). The scatter plot further reveals the correlation between the target gene and various types of immune cells. (**Supplementary Figures 3A–D**). This demonstrated immune microenvironment heterogeneity between risk groups and their relevance to ferroptosis activity.

In summary, the above results suggest that ESCC tumor tissues are characterized by active immune cell infiltration, which may affect tumor progression and immune response, and provide a theoretical basis for the synergistic therapeutic strategy of co-targeting ferroptosis pathway and immune checkpoints.

### Tumor mutational burden drug sensitivity and response to immunotherapy

**Figure 8:**
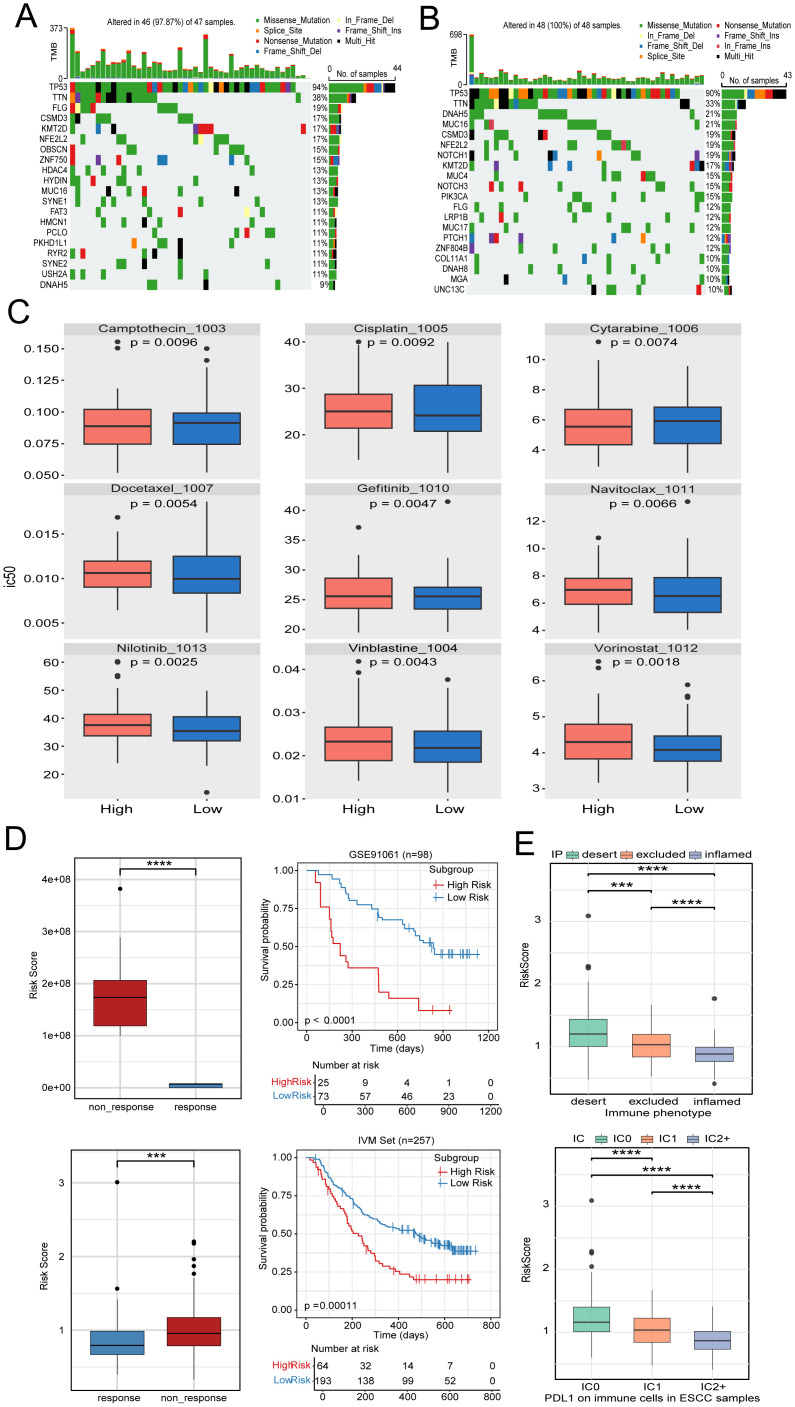
TMB drug sensitivity and response to immunotherapy between high- and low-risk group. **(A)** Waterfall plot of the top 20 genes with the highest mutation frequency in the high-risk group. **(B)** Waterfall plot of the top 20 genes with the highest mutation frequency in the low-risk group. **(C)** Comparison of the IC50 values of 10 chemotherapeutic drugs between the high-risk and low-risk groups. **(D)** Distribution of risk scores in different clinical responses to cancer immunotherapy and survival curves for high- and low-risk expression in PD-L1-treated cohorts in the GSE91061 (n=98) and IMvigor210 (n=257) cohorts **(E)** Differences in risk scores and PD-L1 expression among three phenotypes in Chinese ESCC patients. ***: P<0.001;****: P<0.0001.

To evaluate potential response to immunotherapy across ESCC risk subgroups, tumor mutational burden (TMB) was analyzed alongside specific mutation profiling. Genomic characterization revealed *TP53* as the most frequently altered gene in all cohorts, with *TTN* ranking second in mutation prevalence (**Figures 8A, B**). TMB assessment revealed significant differences between the two groups (**Supplementary Figure 4A**). TMB analysis suggested potential resistance mechanisms and supported combined therapeutic strategies. Additionally, drug sensitivity analysis predicted that high-risk patients may be more sensitive to conventional chemotherapeutic agents like cisplatin and docetaxel, potentially leading to improved therapeutic outcomes with intensified regimens (**Figure 8C**). To further validate the clinical utility of the sub-risk stratification, we assessed its correlation with immunotherapy outcomes. Analysis of the GSE91061 and IMV210 cohorts revealed significantly elevated risk scores in treatment-resistant patients compared to responders (*p* < 0.0001). Consistent with this finding, Kaplan-Meier analysis demonstrated superior survival outcomes in low-risk patients across both cohorts (log-rank *p* < 0.0001), reinforcing the prognostic value of the risk stratification model for immunotherapy response prediction (**Figure 8D**). Finally, the risk score tracked tightly with the tumour immune phenotype: patients mapping to the immune-desert class carried the highest scores, whereas those in the inflammatory class displayed the lowest. Risk values declined linearly with *PD-L1* abundance on immune cells, underscoring an inverse association between *PD-L1* expression and predicted risk. Consistently, high-risk cases showed poor immunotherapy efficacy, while low-risk patients are expected to derive greater clinical benefit (**Figure 8E**).

### Prognostic nomogram of ESCC risk model based on ferroptosis-related genes

**Figure 9:**
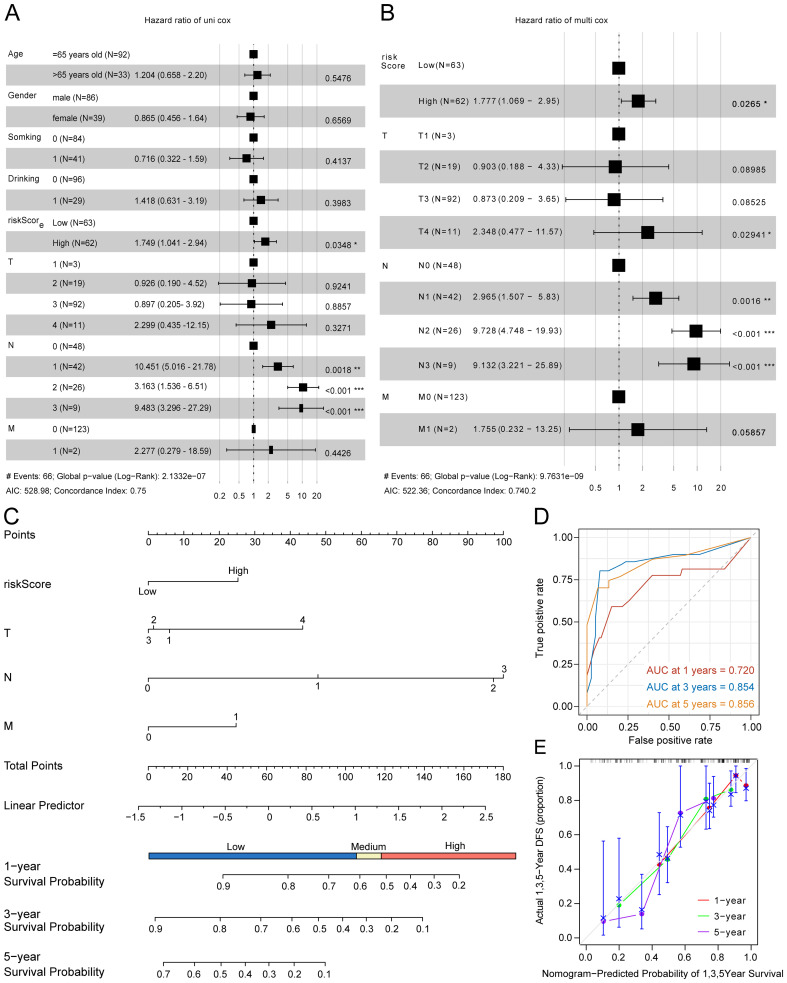
Construction of nomograms for risk scores and clinical features. **(A)** Forest plot of univariate Cox regression analysis. **(B)** Forest plot of multivariate Cox regression analysis. **(C)** Column line plots combining risk scores with clinical characteristics **(D)** Time-dependent ROC curves of the prediction model for 1-year, 3-year, and 5-year survival. **(E)** Calibration curves of the prognostic model for 1-year, 3-year, and 5-year overall survival. *: P<0.05; **: P<0.01; ***: P<0.001.

The prognostic value of the Ferroptosis-Related Genes -based risk model was assessed through univariate and multivariate Cox regression analyses (**Figures 9A, B**; **Supplementary Figure 5A**). These analyses identified the model as an independent prognostic indicator in ESCC patients, with multivariate results confirming its predictive independence. Subsequently, a nomogram was developed based on the model parameters (**Figure 9C**). ROC curve evaluation demonstrated robust predictive performance, with AUC values of 0.72 (1-year), 0.854 (3-year), and 0.856 (5-year), indicating excellent prognostic discrimination capability (**Figures 9D, E**). Overall, the integrated nomogram provides a reliable tool for individualized prognostication and may assist in guiding clinical decision-making.

### *In vitro* validation of key genes (e.g. *CDCA3*)

**Figure 10:**
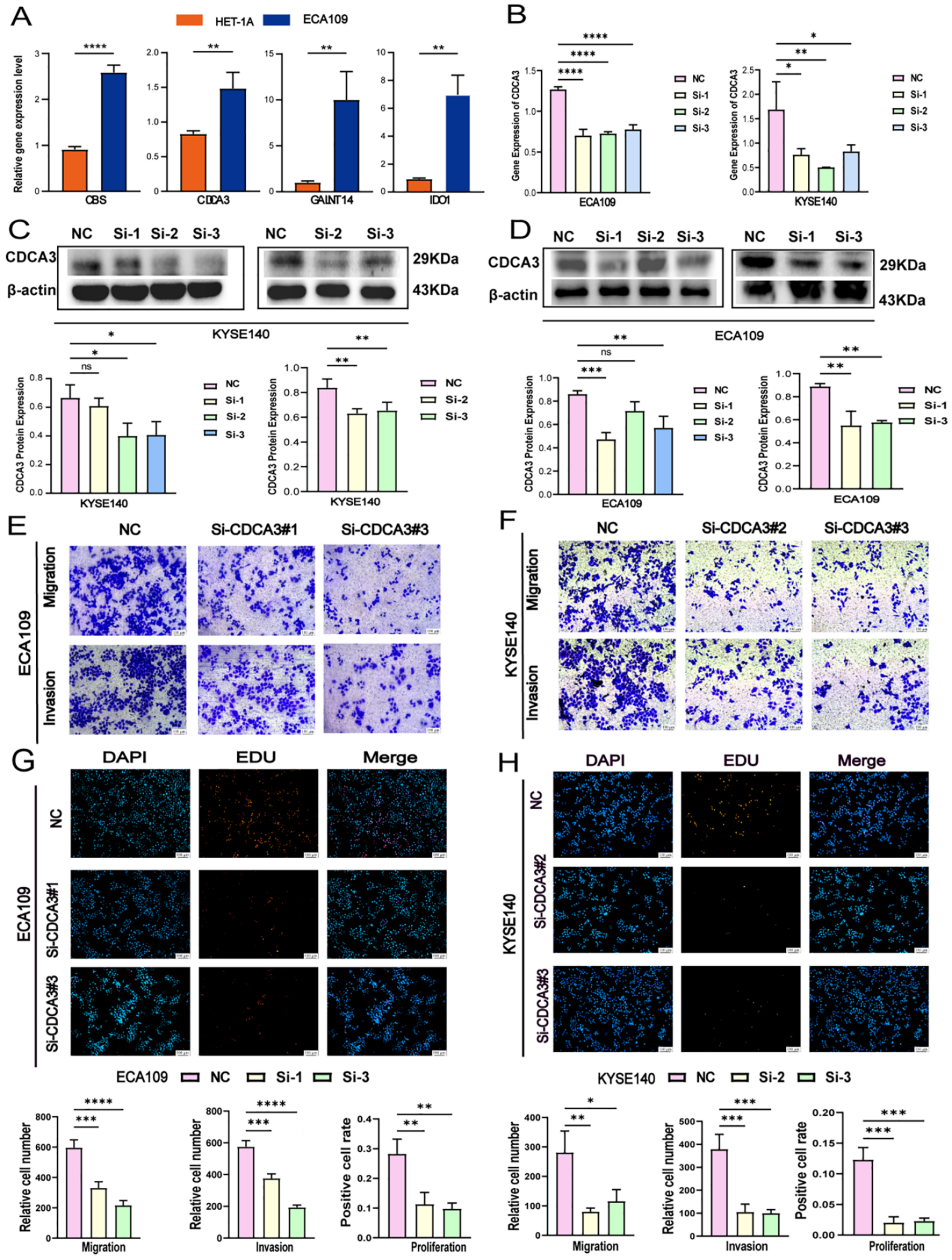
Effects of CDCA3 knockdown on the malignant phenotypes of ESCC cells and ferroptosis. **(A)** qRT-PCR was employed to assess the expression levels of ferroptosis-related markers, including *GALNT14*, *IDO1*, *CDCA3*, and *CBS*, in both normal esophageal epithelial cell lines (HET-1A) and esophageal squamous cell carcinoma (ESCC) cell lines. **(B-D)** Detection of *CDCA3* Knockdown Efficiency in KYSE140 and ECA109 Cells by qRT-PCR and Western Blot. **(E-F)** Transwell Assay for Invasion and Migration in KYSE140 and ECA109 Cells. **(G-H)** EDU assays were performed to evaluate the proliferative capacity of KYSE140 and ECA109 cells. *: P<0.05; **: P<0.01; ***: P<0.001; ****: P<0.0001.

To verify the differential expression of four ferroptosis-related genes between tumor and normal tissues, we first conducted a differential analysis using the TCGA database (**Supplementary Figure 6A**). Subsequently, we further validated the expression differences of these genes through qRT-PCR experiments. The results showed that the expression levels of *CBS*, *CDCA3*, *GALNT14*, and *IDO1* were significantly different compared with normal esophageal epithelial cells (*p* < 0.05), which further confirmed the clinical application value of the model (**Figure 10A**). These validations affirmed the transcriptional and translational significance of model genes in ESCC. First, quantitative real-time PCR and western blot were performed to detect the mRNA and protein expression levels of CDCA3 in normal esophageal epithelial cells (Het-1A) and four esophageal squamous cell carcinoma cell lines (KYSE140, ECA109, TE-13 and TE-1) (**Supplementary Figures 6B, C**). The results showed that *CDCA3* expression in KYSE140 and ECA109 cell lines was significantly higher than that in normal esophageal epithelial cells. Subsequently, a *CDCA3* knockdown model was constructed with small interfering RNAs in KYSE140 and ECA109 cell lines, and the knockdown efficiencies were examined by qRT-PCR and Western Blot, which showed that both interfering sequences were able to effectively reduce the mRNA and protein expression levels of *CDCA3*, so these two sequences were selected for subsequent experiments (**Figures 10B–D**). Cell function experiments further revealed the effects of *CDCA3* knockdown on the behavior of ESCC cells. Transwell assay results showed that knockdown of *CDCA3* significantly inhibited the invasion and migration ability of ESCC cells (**Figures 10E, F**). In addition, CCK8 and EDU assays showed that *CDCA3* knockdown significantly inhibited the proliferation ability of ESCC cells (**Figures 10G, H**, **Supplementary Figure 6D**). Collectively, these results indicate that CDCA3 functions as an oncogene to positively regulate the malignant phenotypes of proliferation, invasion and migration in esophageal squamous cell carcinoma cells.

### External validation of gene signatures in GSE188900 and esophageal cancer tissues

**Figure 11:**
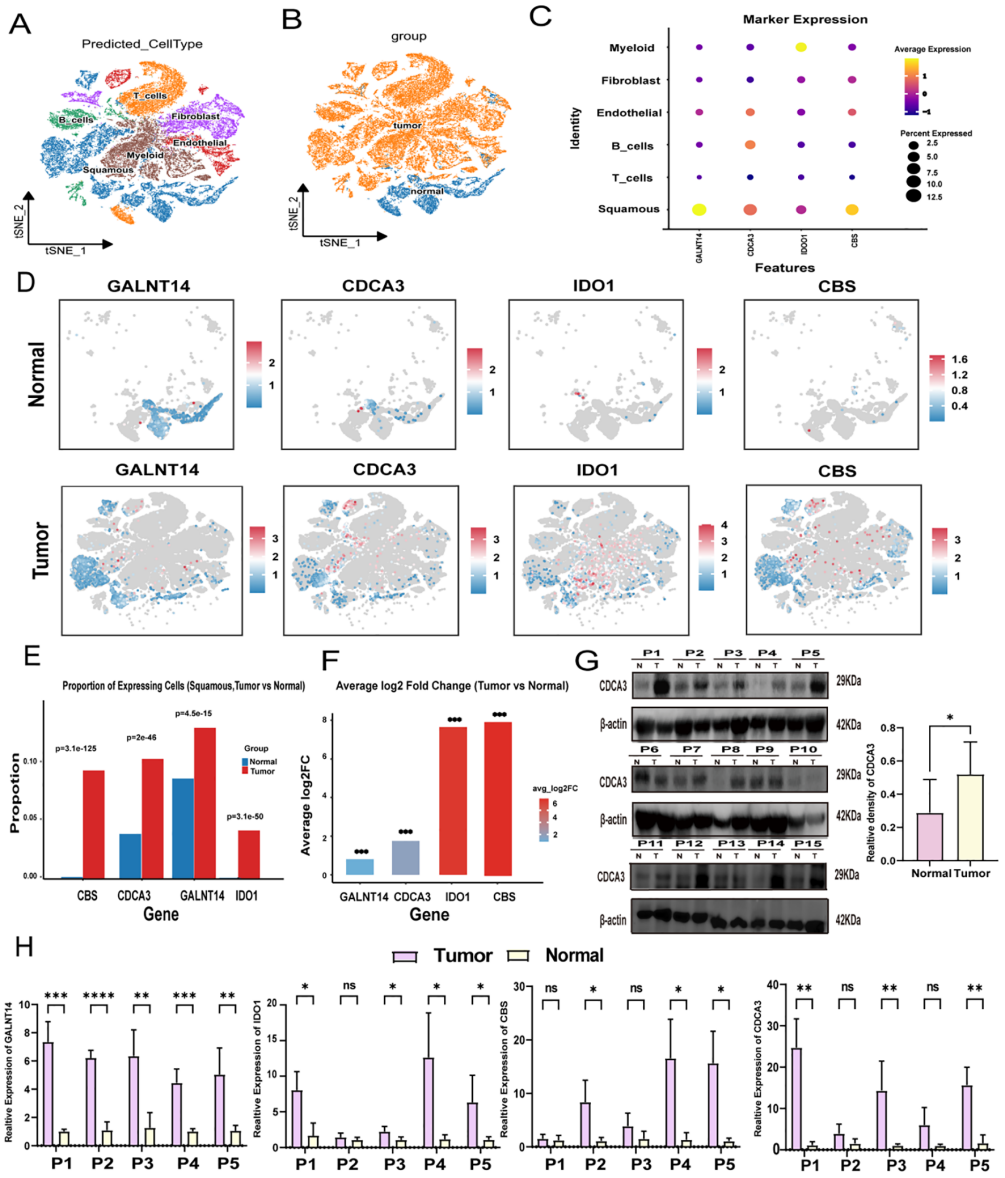
The expression of ferroptosis-related genes was validated in the GSE188900 dataset and Esophageal cancer patient tissues. **(A)** The t-SNE map of the single-cell ESCC landscape was colored by cell subtypes. **(B)** The t-SNE map showed the distribution of epithelial cells from normal and tumor tissues in the GSE188900. **(C)** A bubble chart displayed the expression levels of ferroptosis genes across different cell subtypes. **(D)** The t-SNE map illustrates the distribution of ferroptosis-related genes in tumor and normal tissues. **(E, F)** The expression levels of ferroptosis genes in epithelial cells from normal esophagus and tumor tissues were shown in the data. **(G)** The protein expression of *CDCA3* in 15 pairs of esophageal squamous cell carcinoma patient tumor tissues and adjacent non-tumor tissues. **(H)** The mRNA expression of *GALNT14*, *IDO1*, *CDCA3*, and *CBS* in 5 pairs of ESCC patient tumor tissues and adjacent non-tumor tissues. *: P<0.05; **: P<0.01; ***: P<0.001; ****: P<0.0001.

To confirm the involvement of the target genes in ESCC, we further validated them using the GSE188900 dataset. First, we annotated the cells into six types—T cells, Fibroblasts, myeloid cells, B cells, Endothelial cells, and Squamous cells—based on marker genes. The cell clusters were then classified as normal or tumor (**Figures 11A, B**). The results showed that the expression of *CBS*, *GALNT14*, *CDCA3*, and *IDO1* was significantly higher in esophageal cancer cells compared to normal esophageal cells (**Figures 11C–F**). Subsequently, we obtained 15 pairs of esophageal cancer tissues and their adjacent non-cancerous counterparts from patients with esophageal cancer at the Affiliated Tumor Hospital of Xinjiang Medical University. Among them, 15 pairs were used for Western blot analysis, and 5 pairs were subjected to qRT-PCR to further validate the expression levels of the target genes. The results showed that the expression of *CDCA3* was significantly higher in cancer tissues than in adjacent non-cancerous tissues at both the protein and mRNA levels (**Figures 11G, H**). This finding further confirms the potential clinical value of model in the diagnosis of esophageal cancer.

### Knockdown of CDCA3 enhances RSL3-induced ferroptosis

**Figure 12:**
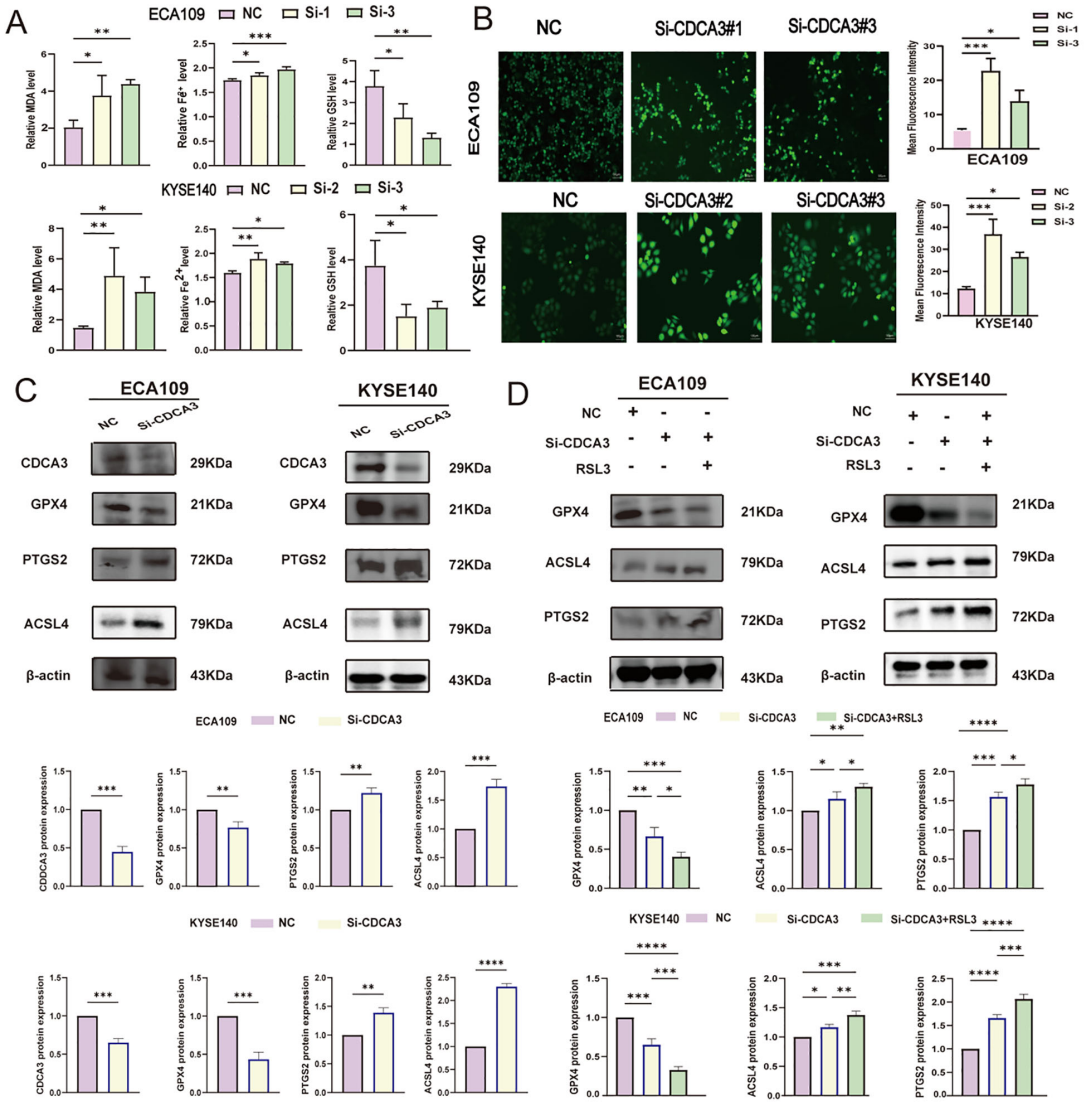
Knockdown of CDCA3 promotes ferroptosis. **(A, B)** Malondialdehyde, Ferrous ions, Glutathione and Reactive oxygen species content. **(C)**. CDCA3 Knockdown alters the expression levels of GPX4,PTGS2 and ACSL4 **(D)**. CDCA3 Knockdown promotes RSL3 induced ferroptosis. *: P<0.05; **: P<0.01; ***: P<0.001; ****: P<0.0001.

To investigate the role of CDCA3 in ferroptosis regulation, In the *in vitro* cell model, an oxidative stress assay kit and flow cytometry experiments were employed to investigate the changes in malondialdehyde (MDA), glutathione (GSH), ferrous ions (Fe^2+^), and reactive oxygen species (ROS) in esophageal cancer cell lines KYSE140 and ECA109 following *CDCA3* knockdown. The results revealed that compared with the negative control group, *CDCA3* knockdown significantly elevated the levels of Fe2^+^and MDA in esophageal squamous cell carcinoma cells, while markedly reducing the level of GSH. Additionally, oxidative stress assay kit analysis indicated that the ROS content in the *CDCA3* knockdown group was significantly higher than that in the negative control group (**Figures 12A, B**). These changes are typical of ferroptosis, suggesting that *CDCA3* knockdown may inhibit the malignant progression of ESCC cells by inducing oxidative stress and ferroptosis. Subsequently, we established CDCA3 knockdown models in ECA109 and KYSE140 cell lines and examined the expression levels of ferroptosis-related proteins. The findings revealed that CDCA3 knockdown significantly reduced GPX4 protein expression, whereas PTGS2 and ACSL4 levels were markedly upregulated, suggesting that CDCA3 is involved in the regulation of ferroptosis in esophageal cancer cells(**Figure 12C**). To further elucidate the effect of CDCA3 on ferroptosis, we treated CDCA3-knockdown cells with the ferroptosis inducer RSL3. The results indicated that compared to the control group (NC group) or cells with only CDCA3 knockdown, the combination of CDCA3 knockdown and RSL3 treatment led to a further decrease in GPX4 expression and a significant increase in PTGS2 and ACSL4 expression, demonstrating that CDCA3 suppression enhances the ability of RSL3 to induce ferroptosis, thereby promoting its occurrence(**Figure 12D**).

In summary, the present study revealed the critical role of *CDCA3* in ESCC cell proliferation, migration and ferroptosis through *in vitro* experiments, which provides a new potential target for the treatment of ESCC.

## Discussion

Esophageal squamous cell carcinoma (ESCC) remains a highly intractable malignancy, where poor survival outcomes stem from late diagnosis, aggressive invasion, frequent recurrence, and intrinsic to platinum-based chemotherapy-especially in *TP53*-mutant subtypes (13, 14). Previous studies have established multiple prognostic models for esophageal squamous cell carcinoma based on ferroptosis-related genes. For instance, Lu and Song et al (15, 16). developed such models using transcriptomic data. However, most of these models rely on large-scale transcriptomic datasets and lack in-depth analysis at the single-cell level. Furthermore, although attempts have been made to construct prognostic models at the single-cell level, the absence of independent external single-cell datasets for validation has limited their generalizability and clinical applicability (17).In this study, we integrated single-cell transcriptomic data to dissect the heterogeneity of the tumor microenvironment, constructed a four-gene signature for prognosis, and conducted systematic *in vitro* functional validation of the core gene CDCA3. Compared to existing prognostic biomarker systems, this signature enhances the accuracy of prognostic stratification in ESCC patients and reveals previously underexplored crosstalk between the ferroptosis pathway and the tumor immune microenvironment, thereby providing insights for mechanistic studies of precision immunotherapy (18–20). Further analysis using immune checkpoint therapy patient cohorts demonstrated its ability to distinguish treatment response differences among ferroptosis risk subgroups, highlighting its potential translational value in personalized immune interventions. Additionally, the prognostic model was validated using both public independent single-cell datasets and clinical ESCC tissue samples, further supporting its reliability for clinical application.

Notably, our analysis uncovered a paradoxical immune landscape in high ferroptosis-risk ESCC subsets, characterized by a dynamic equilibrium in which immune activation and suppression coexist. Elevated frequencies of CD4+ memory T cells and activated dendritic cells suggest ongoing immunosurveillance, yet this is counterbalanced by the enrichment of M0 macrophages and the immunosuppressive enzyme *IDO1*, which together facilitate tumor immune escape (21, 22). This dualistic phenomenon mirrors observations in other solid tumors, such as hepatocellular carcinoma and melanoma, where ferroptosis-induced lipid peroxidation enhances immunogenic cell death while simultaneously upregulating checkpoint molecules that suppress T cell function (23). Among the model’s constituent genes, *IDO1* emerges as a critical node linking ferroptosis to immune dysfunction: by catalyzing tryptophan degradation into kynurenine, it induces T cell anergy and promotes regulatory T cell recruitment, mechanisms previously validated in glioblastoma and colorectal cancer (21). Concurrently, *CBS*—an enzyme involved in hydrogen sulfide metabolism—modulates redox balance to influence ferroptosis sensitivity while interfering with T cell infiltration via SIRT1-dependent pathways in gastrointestinal tumors (24), establishing a bidirectional feedback loop between ferroptosis and immune evasion.

Our CellChat-based analysis further uncovers enhanced signaling through LAMININ and MHC-I pathways in ferroptosis-high subgroups, highlighting structural and immunological remodeling of the TIME. As core components of the extracellular matrix (ECM), LAMININ promote tumor progression via integrin-mediated adhesion and Notch signaling (25), with their enrichment in epithelial-monocyte-endothelial communication networks suggesting that ferroptosis-driven epithelial remodeling creates an invasion-permissive microenvironment. Concurrently, aberrant activation of MHC-I by stressed epithelial cells may facilitate immune editing and T cell exhaustion—a phenomenon conserved in ferroptosis-enriched (26)—thereby explaining the limited immunotherapeutic responses observed in high-risk subsets.

Gene set enrichment analysis (GSEA) identifies PI3K-AKT and ECM-receptor interaction pathways as central to the high-risk phenotype, linking ferroptosis to oncogenic and mechanotransductive programs. *CDCA3*, a core model gene, activates the PI3K-AKT axis to confer proliferative advantages and resistance to ferroptosis-induced cell death (27, 28), a vulnerability exploited in ovarian and colorectal cancer through PI3K-AKT inhibition to restore ferroptosis sensitivity and enhance immunotherapy response. *GALNT14*, a glycosyltransferase included in the model, promotes ferroptosis via EGFR/mTOR pathway inhibition in ovarian cancer (29), yet its role in ESCC may involve dual regulation of lipid peroxidation and cell surface glycoprotein modifications that influence immune cell recognition—an emerging axis with profound implications for tumor-immune crosstalk.

The relationship between ferroptosis and tumor mutational burden (TMB)—a validated biomarker of immunotherapy responsiveness—adds another layer of complexity (30). While ferroptosis-related stress may induce genomic instability and neoantigen accumulation via lipid peroxidation-induced replication stress, high ferroptosis-risk ESCC patients in our cohort exhibited lower predicted immunotherapy efficacy despite evidence of T cell activation. This paradox likely arises from compensatory immune evasion mechanisms, such as impaired antigen presentation or enhanced expression of immunosuppressive molecules, which overshadow the potential benefits of increased TMB.

Taken together, our findings position ferroptosis regulators as central orchestrators of a complex signaling network that intersects with ECM dynamics, immune microenvironment stability, and heterogeneous treatment outcomes in ESCC. The dysregulation of these genes not only drives tumor progression but also contributes to the structural and immunological architecture of the TIME, offering a mechanistic explanation for the poor prognosis associated with high ferroptosis risk. Spatial transcriptomics and single-cell proteomics will further illuminate cell-cell interaction dynamics *in situ*, while external validation across ethnically and clinically diverse cohorts is critical to ensure generalizability.

It is important to note that the GSE91061 dataset and the IMvigor210 cohort used for immunotherapy response analysis are not specific to esophageal squamous cell carcinoma. These datasets primarily consist of samples from other epithelial-derived malignancies, and lack dedicated transcriptomic and clinical response data from ESCC patients undergoing immunotherapy. Therefore, the association between CDCA3 and immunotherapy sensitivity identified based on these cohorts cannot be directly extrapolated to ESCC. Future studies should collect clinical specimens and follow-up data from ESCC patients who have received immunotherapy, and conduct cohort validation to further clarify the potential predictive value of CDCA3 in the response to immunotherapy in esophageal squamous cell carcinoma.In addition, single-cell sequencing may introduce selection bias. In the future, we will conduct multidimensional validation through enlarged samples and spatial transcriptome technology (Visium HD) to further improve the spatiotemporal map of the ESCC ferroptosis regulatory network.

## Conclusions

In summary, our study presents a single-cell resolved ferroptosis risk model that redefines prognostic stratification in ESCC by integrating cellular heterogeneity, signaling pathways, and immune modulation. By uncovering ferroptosis as a nexus linking ECM reprogramming, immune evasion, and therapy resistance, these findings provide a foundation for developing stratified therapeutic strategies—such as *IDO1* inhibition in ferroptosis-high subtypes or PI3K-AKT targeting to resensitize resistant tumors. Beyond ESCC, this work underscores the power of single-cell multi-omics to unravel cancer complexity, offering a translational blueprint for precision oncology approaches that harmonize ferroptosis biology with immunotherapy.

## Data Availability

In this study, transcriptome data from 125 cases were obtained from the Xinjiang Medical University Affiliated Tumor Hospital. The original transcriptome and clinical data have been deposited in the GSA-Human database (HRA000178) at the China National Center for Bioinformation/Beijing Institute of Genomics, Chinese Academy of Sciences, and are available at https://ngdc.cncb.ac.cn/gsa-human. We also utilized data from other public sources, including The TCGA (https://www.cancer.gov/tcga) and GSE53624 (http://www.ncbi.nlm.nih.gov/geo). All relevant data will be shared by the corresponding authors upon reasonable request.
