# Supplementary figures and images for: Single-cell-derived ferroptosis signature predicts prognosis and therapy response in esophageal squamous cell carcinoma
Source: Front Oncol. 2026 Jul 17;16:1873687. doi: 10.3389/fonc.2026.1873687 (PMC13423694; doi:10.3389/fonc.2026.1873687)

orig.ident

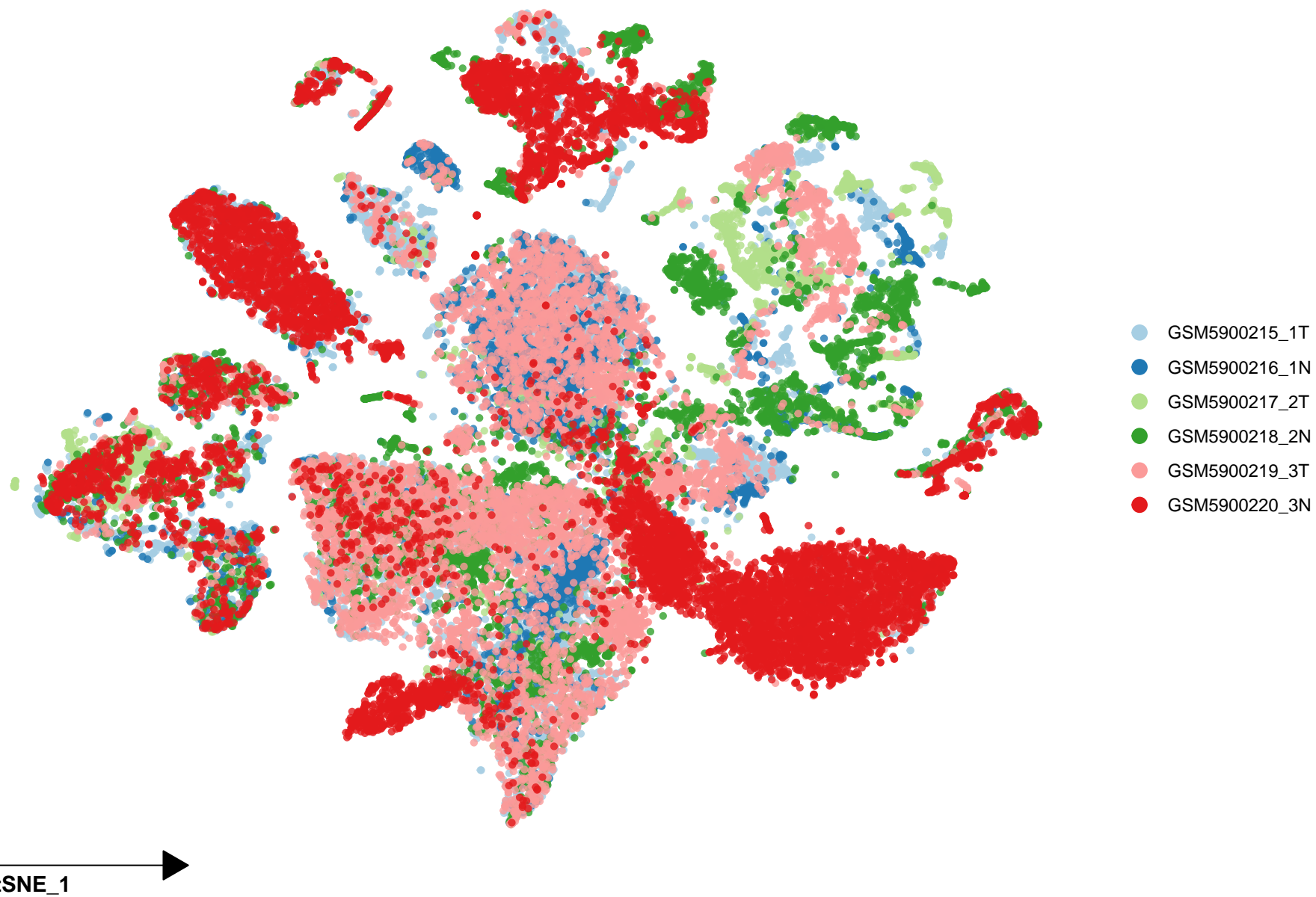

Supplement: Supplementary file 1 [file DataSheet1.pdf]

**nFeature\_RNA**

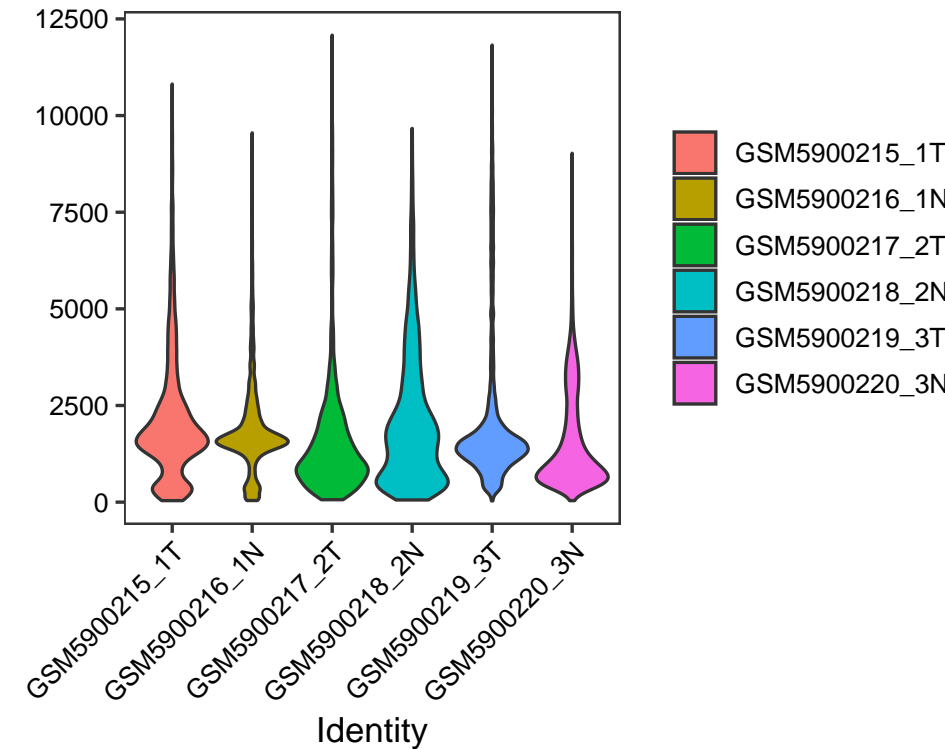

**nCount\_RNA**

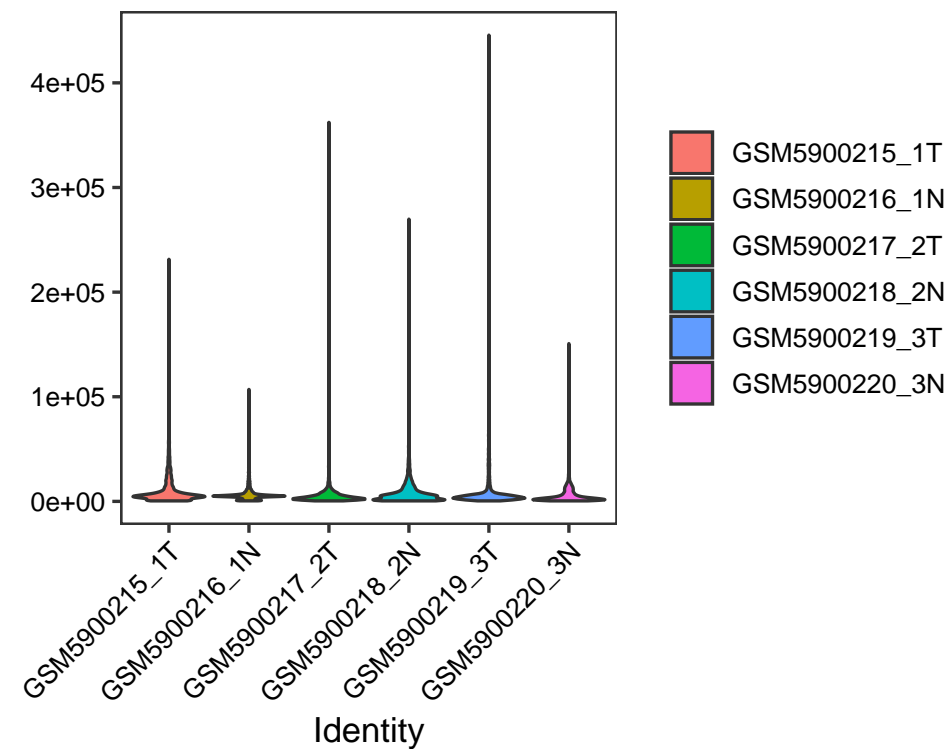

**percent.mt**

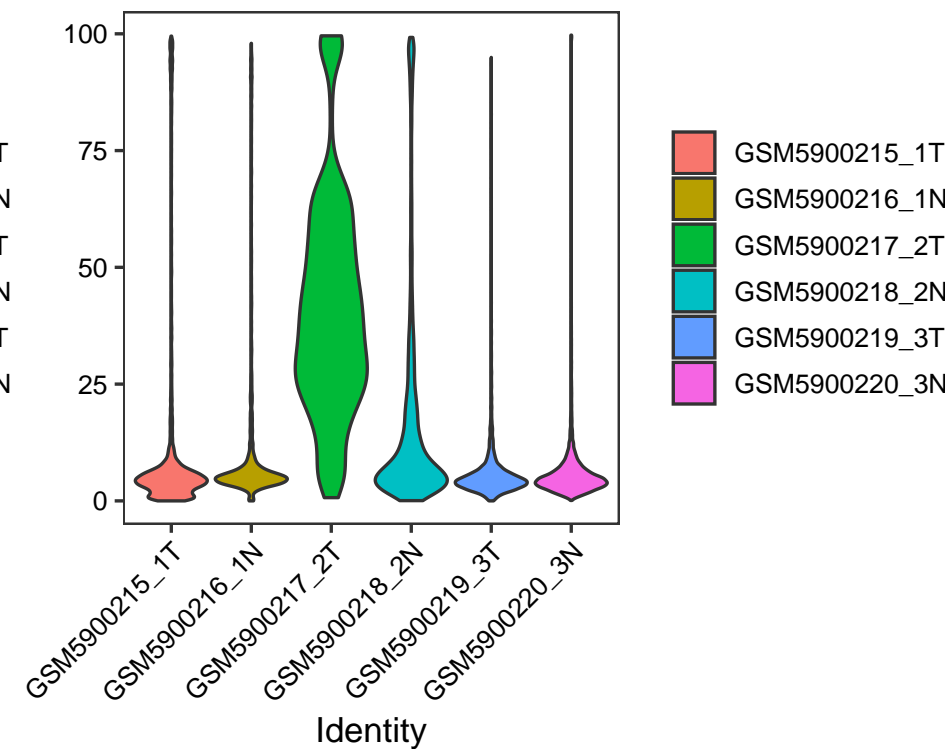

**percent\_mito**

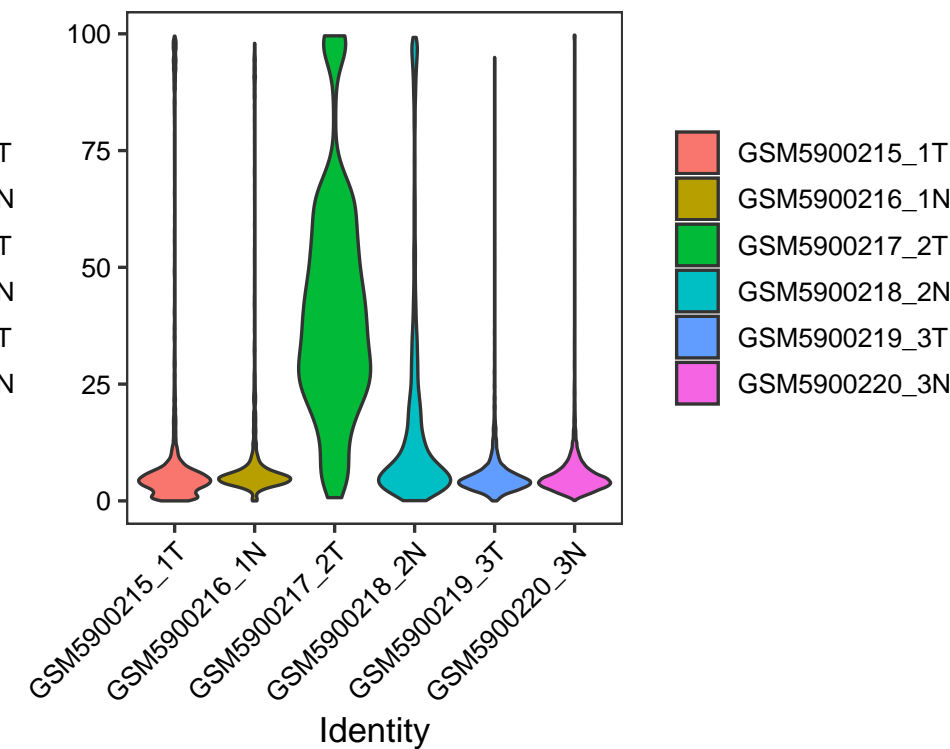

Supplement: Supplementary file 2 [file DataSheet2.pdf]

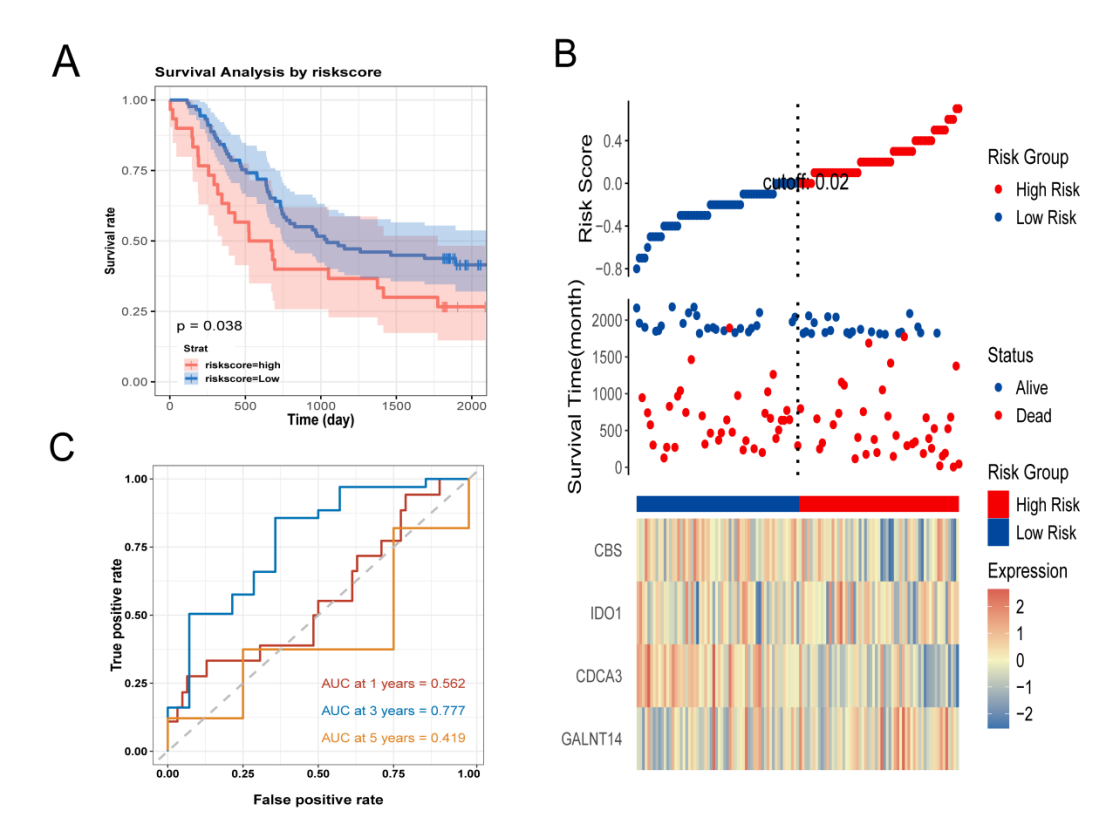

Supplement: Supplementary file 3 [file Image1.png]

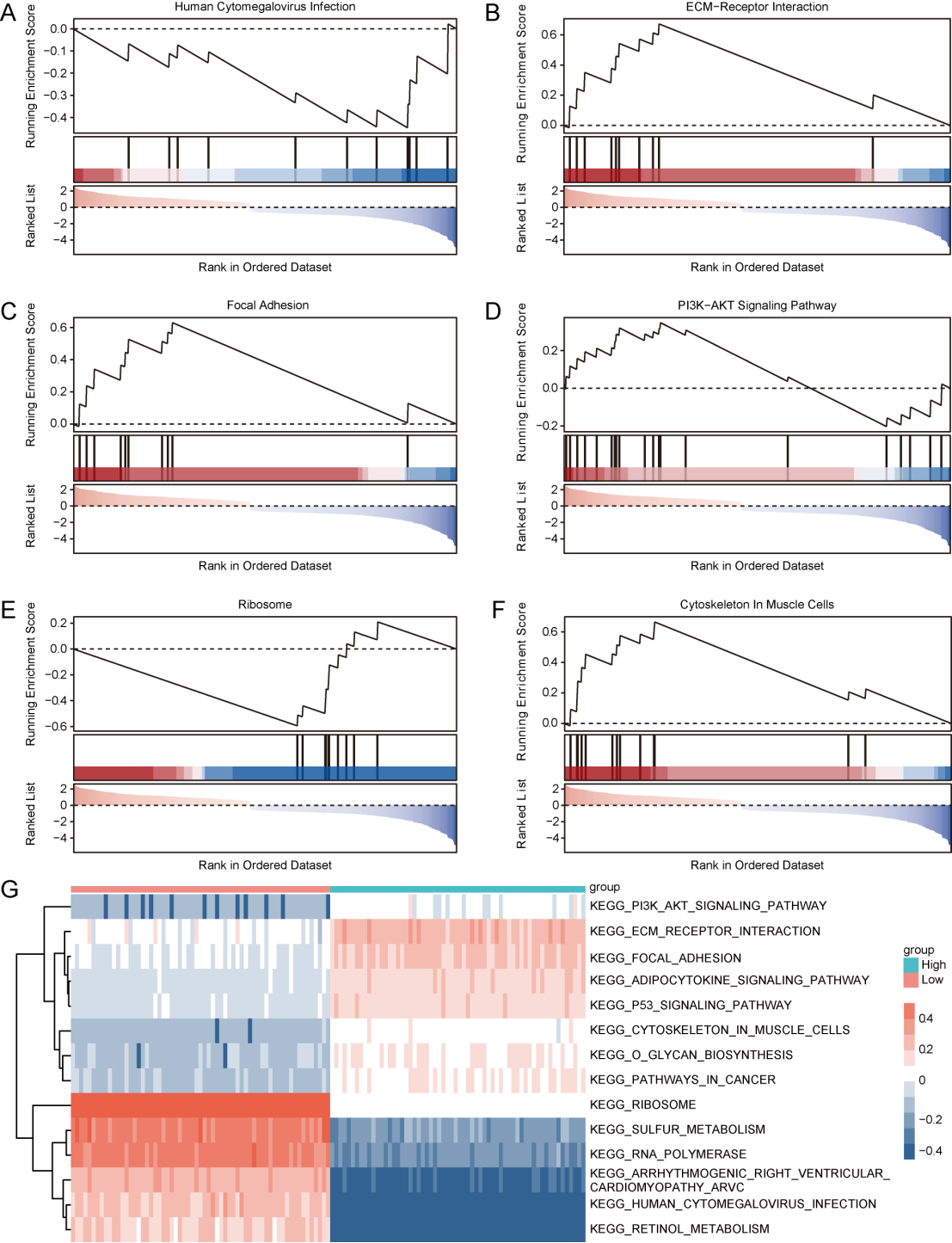

Supplement: Supplementary file 4 [file Image2.png]

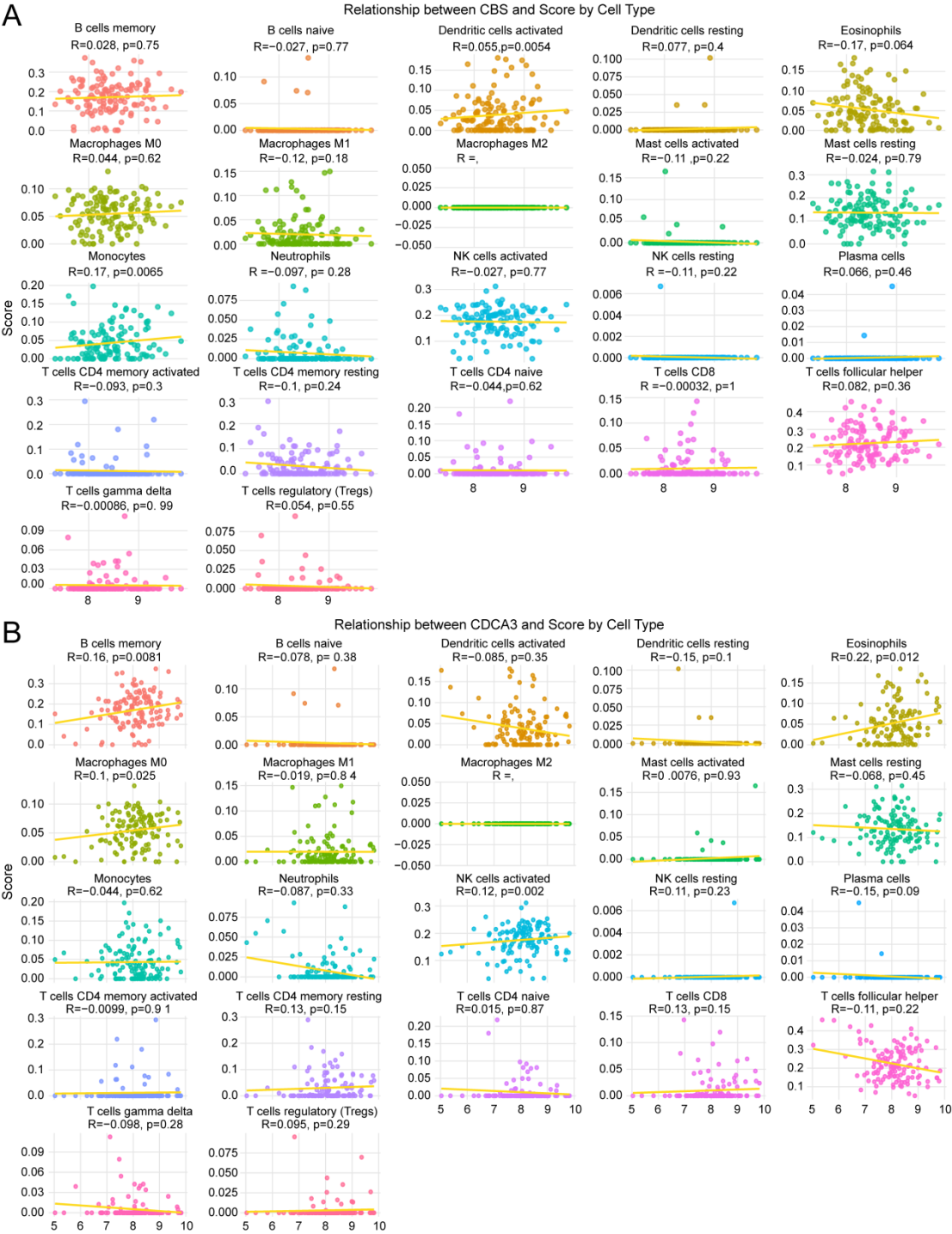

Supplement: Supplementary file 5 [file Image3.png]

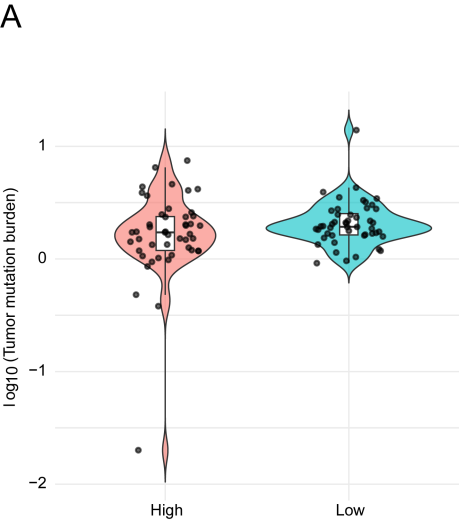

Supplement: Supplementary file 6 [file Image4.png]

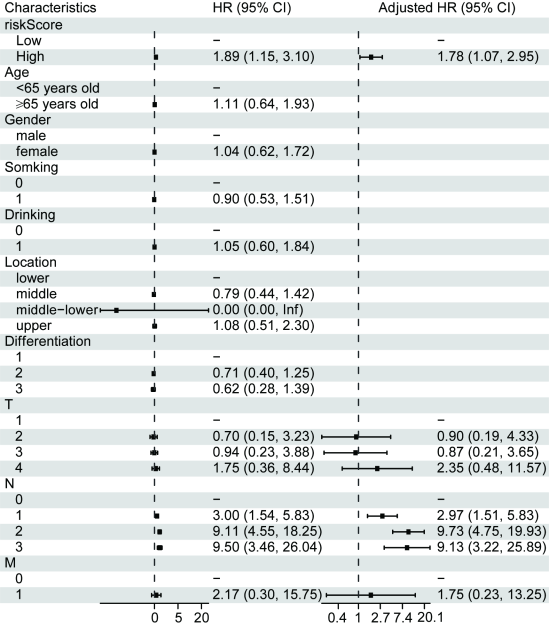

Supplement: Supplementary file 7 [file Image5.png]

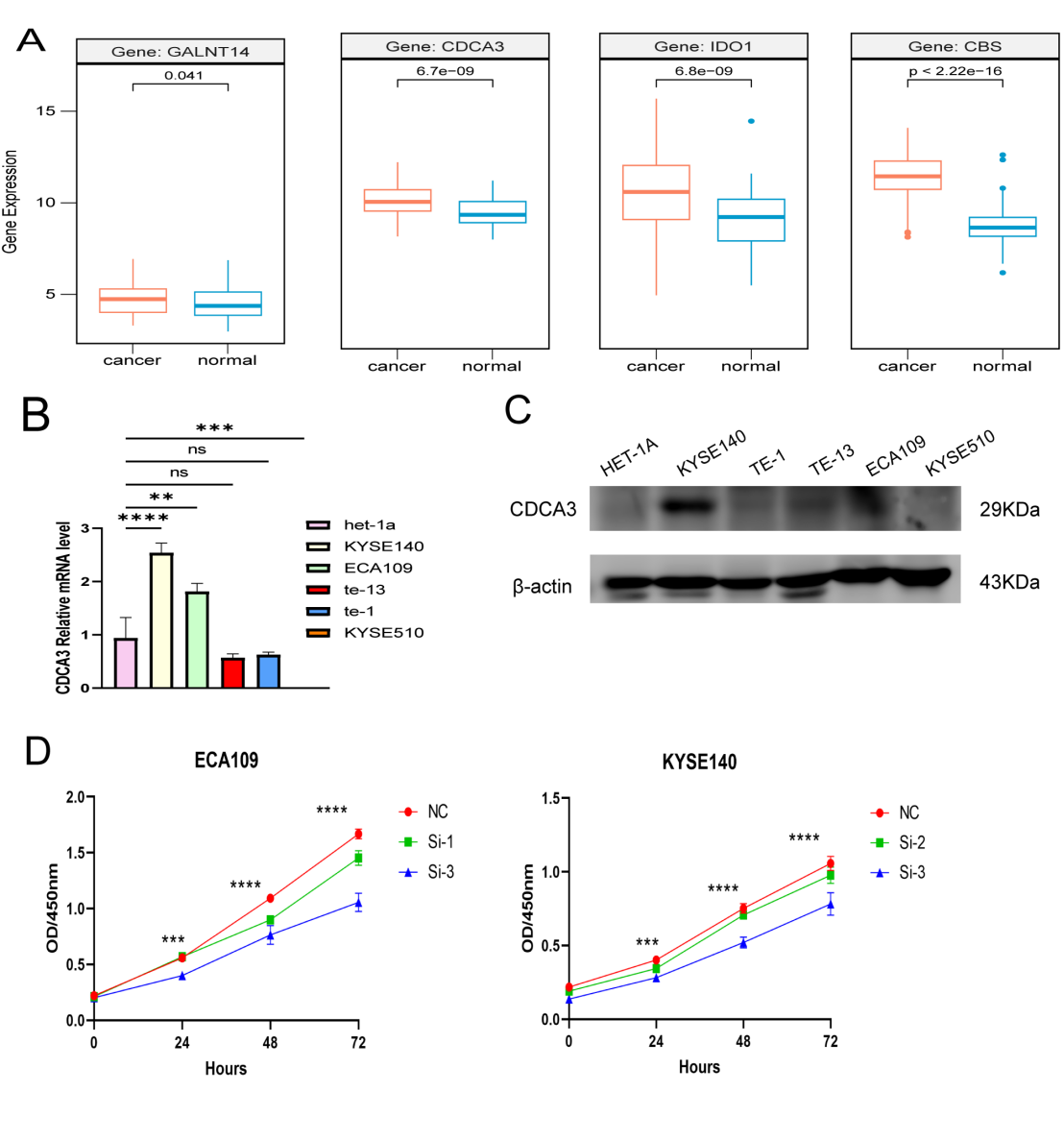

Supplement: Supplementary file 8 [file Image6.png]
