# Supplementary material for: Single-cell-derived ferroptosis signature predicts prognosis and therapy response in esophageal squamous cell carcinoma
Source: Front Oncol. 2026 Jul 17;16:1873687. doi: 10.3389/fonc.2026.1873687 (PMC13423694; doi:10.3389/fonc.2026.1873687)
Supplement: Supplementary file 9 [file Table1.docx]

| **Antibodies and Reagents** | **Brand** | **Catalog No** |
| --- | --- | --- |
| CDCA3 Antibody | Affinity | #DF3544 (1:1000) |
| GPX4 | Proteintech | 30388-1-AP(1:1000) |
| PTGS2 | Proteintech | 27308-1-AP(1:1000) |
| ACSL4 | Proteintech | 22401-1-AP(1:1000) |
| β-actin | Boster | BM0627(1:10000) |
| RSL3 | MCE | HY-100218A |
| Si-NC | Heyuan Biology | **F：**UUCUCCGAACGUGUCACGUTT  **R：**ACGUGACACGUUCGGAGAATT |
| Si-CDCA3-1 | Heyuan Biology | **F：**CCAGUUAUCUGUUGAGGAATT  **R：**UUCCUCAACAGAUAACUGGTT |
| Si-CDCA3-2 | Heyuan Biology | **F：**GCAAGACAGCCCACAGAAATT  **R：**UUUCUGUGGGCUGUCUUGCTT |
| Si-CDCA3-3 | Heyuan Biology | **F：**GCACCUUUAUCUUCUGAAUTT  **R：**AUUCAGAAGAUAAAGGUGCTT |
| BeyoClickEDU-555Cell Proliferation Kit | Beyotime | C00755 |
| MDAContent Detection Kit | Solibao | BC0025 |
| GSH Content Detection Kit | Beyotime | C5003 |
| Fe^2+^Content Detection Kit | Solibao | BC5415 |
| ROS Content Detection Kit | Solibao | CA1410 |
