# Supplementary material for: Single-cell-derived ferroptosis signature predicts prognosis and therapy response in esophageal squamous cell carcinoma
Source: Front Oncol. 2026 Jul 17;16:1873687. doi: 10.3389/fonc.2026.1873687 (PMC13423694; doi:10.3389/fonc.2026.1873687)
Supplement: Supplementary file 10 [file Table2.docx]

**Supplementary table 2**. Baseline characteristics of ESCC tissue samples used for Western blot analysis.

| Clinicopathological  Parameters | Categories | Number of cases (n=15) | Percentage (%) |
| --- | --- | --- | --- |
| Age (years) |  |  |  |
|  | ＞60 | 9 | 60% |
|  | ≤60 | 6 | 40% |
| Gender |  |  |  |
|  | Male | 11 | 73% |
|  | Female | 4 | 27% |
| differentiation grade |  |  |  |
|  | High | 3 | 20% |
|  | Middle | 5 | 33% |
|  | Low | 7 | 47% |
| Gross morphology |  |  |  |
|  | Ulcerative | 6 | 40% |
|  | Fungating | 0 | 0% |
|  | Medullary | 5 | 33% |
|  | Protruding | 4 | 27% |
| Tumor maximum diameter (cm) |  |  |  |
|  | <3 | 4 | 27% |
|  | 3–5 | 8 | 53% |
|  | >5 | 3 | 20% |
| T stage (Tumor invasion depth) |  |  |  |
|  | T1 | 2 | 13% |
|  | T2 | 4 | 27% |
|  | T3 | 4 | 27% |
|  | T4 | 5 | 33% |
| N stage (Lymph node metastasis) |  |  |  |
|  | N0 | 1 | 7% |
|  | N1 | 5 | 33% |
|  | N2 | 6 | 40% |
|  | N3 | 3 | 20% |
| M stage (Distant metastasis) |  |  |  |
|  | No distant metastasis | 10 | 67% |
|  | With distant metastasis | 5 | 33% |
| TNM Stage |  |  |  |
|  | I | 3 | 20% |
|  | II | 5 | 33% |
|  | III | 7 | 47% |
| Survival outcome |  |  |  |
|  | Survival | 4 | 27% |
|  | Death | 11 | 73% |
